# The importance of murine phospho-MLKL-S345 in situ detection for necroptosis assessment in vivo

**DOI:** 10.1038/s41418-024-01313-6

**Published:** 2024-05-23

**Authors:** Konstantinos Kelepouras, Julia Saggau, Ana Beatriz Varanda, Matea Zrilic, Christine Kiefer, Hassan Rakhsh-Khorshid, Ina Lisewski, Iratxe Uranga-Murillo, Maykel Arias, Julian Pardo, Wulf Tonnus, Andreas Linkermann, Alessandro Annibaldi, Henning Walczak, Gianmaria Liccardi

**Affiliations:** 1 Genome Instability, Inflammation and Cell Death Laboratory, Institute of Biochemistry I, Centre for Biochemistry, Faculty of Medicine, University of Cologne, 50931 Cologne, Germany; 2 Center for Molecular Medicine Cologne (CMMC), University of Cologne, 50931 Cologne, Germany; 3 Cell Death, Inflammation and Immunity Laboratory, CECAD Cluster of Excellence, University of Cologne, 50931 Cologne, Germany; 4 Cell Death, Inflammation and Immunity Laboratory, Institute of Biochemistry I, Centre for Biochemistry, Faculty of Medicine, University of Cologne, 50931 Cologne, Germany; 5 Department of Microbiology, Radiology, Paediatry and Public Heath, Faculty of Medicine, University of Zaragoza/IIS, Aragon, Spain; 6 Centro de Investigacion Biomedica en Red de Enfermedades infecciosas (CIBERINFEC), Instituto de Salud Carlos III, Madrid, Spain; 7 Division of Nephrology, Department of Internal Medicine 3, University Hospital Carl Gustav Carus at the Technische Universität Dresden, Dresden, Germany; 8 Division of Nephrology, Department of Medicine, Albert Einstein College of Medicine, Bronx, NY USA; 9 Centre for Cell Death, Cancer and Inflammation, UCL Cancer Institute, University College London, WC1E 6BT London, UK

**Keywords:** Cell death and immune response, Acute inflammation, Infectious diseases

## Abstract

Necroptosis is a caspase-independent modality of cell death implicated in many inflammatory pathologies. The execution of this pathway requires the formation of a cytosolic platform that comprises RIPK1 and RIPK3 which, in turn, mediates the phosphorylation of the pseudokinase MLKL (S345 in mouse). The activation of this executioner is followed by its oligomerisation and accumulation at the plasma-membrane where it leads to cell death via plasma-membrane destabilisation and consequent permeabilisation. While the biochemical and cellular characterisation of these events have been amply investigated, the study of necroptosis involvement in vivo in animal models is currently limited to the use of *Mlkl*^−/−^ or *Ripk3*^−/−^ mice. Yet, even in many of the models in which the involvement of necroptosis in disease aetiology has been genetically demonstrated, the fundamental in vivo characterisation regarding the question as to which tissue(s) and specific cell type(s) therein is/are affected by the pathogenic necroptotic death are missing. Here, we describe and validate an immunohistochemistry and immunofluorescence-based method to reliably detect the phosphorylation of mouse MLKL at serine 345 (pMLKL-S345). We first validate the method using tissues derived from mice in which Caspase-8 (Casp8) or FADD are specifically deleted from keratinocytes, or intestinal epithelial cells, respectively. We next demonstrate the presence of necroptotic activation in the lungs of SARS-CoV-infected mice and in the skin and spleen of mice bearing a Sharpin inactivating mutation. Finally, we exclude necroptosis occurrence in the intestines of mice subjected to TNF-induced septic shock. Importantly, by directly comparing the staining of pMLKL-345 with that of cleaved Caspase-3 staining in some of these models, we identify spatio-temporal and functional differences between necroptosis and apoptosis supporting a role of RIPK3 in inflammation independently of MLKL versus the role of RIPK3 in activation of necroptosis.

## Introduction

Necroptosis is a caspase-independent form of regulated cell death whose untoward activation drives loss of tissue homoeostasis during development as well as the pathogenesis of many inflammatory and infectious diseases [1]. Necroptosis can be induced in response to the activation of death receptors (DRs) [1, 2], (i.e., the tumour necrosis factor (TNF) receptor superfamily (TNFR-SF) members TNFR1 [3–5], FAS/CD95 [6–8], TRAIL-R1/DR4 and TRAIL-R2/DR5 [9–11]), as well as pattern recognition receptors (PRRs) including toll-like receptor 3 (TLR3) and TLR4 [12–14]. The downstream events elicited by the activation of DRs or PRRs lead to assembly of the necrosome, a cytosolic complex canonically formed by the association of Receptor-Interacting Protein Kinase 1 (RIPK1) and 3 (RIPK3) via their RIP homotypic interaction motif (RHIM) domains [15]. This complex has also been shown to form via the association of the cytosolic DNA and RNA sensor, ZBP1, with RIPK3, however, only in absence of RIPK1 expression or of its RHIM domain (*RIPK1*^*ΔRHIM*^) [16]. The activation of this complex culminates in the phosphorylation, ubiquitination and oligomerisation of the pseudokinase Mixed Lineage Kinase Domain-Like (MLKL), responsible for membrane destabilisation and consequent pore formation necessary for the execution of necroptosis and release of the inner cellular content [1, 15, 17–21]. The activation of MLKL relies on RIPK3-mediated phosphorylation of threonine 357/serine 358 (T357/S358) in human and of serine 345 (S345) in murine MLKL [1, 22–24]. Whilst other phosphorylation events have been described for both human and mouse MLKL by other kinases, the above-mentioned are recognised to be the sole, so far discovered, mediated by RIPK3 and to be responsible for the execution of necroptosis [23–25]. Currently, these are widely utilised as conclusive markers to detect this specific cell death modality, however, their utilisation has been limited to western blotting and/or immunofluorescence in adherent cells [26]. To date, there is no reliable and reproducible way of addressing necroptosis in situ in tissues derived from different animal models. The assessment of cell death in vivo relies on detection of cleaved Caspase-3 (c-Casp3) [27] or so called TUNEL (terminal deoxynucleotidyl transferase dUTP nick end labelling) staining (a hallmark of intrinsic and extrinsic apoptosis but also necrosis, necroptosis, pyroptosis and parthanatos [28–33]). Yet, neither are they specific to necroptosis execution nor can they discriminate between different modalities of cell death and MLKL-driven necroptosis. Being able to differentiate between distinct modalities of cell death impacts profoundly on the molecular understanding of the biochemical process responsible for a given pathological condition and importantly, allows (a) the possibility to test pharmacologically targeting of a specific cell death mechanism in an informed way, (b) the discovery and prediction of necroptotic cell death involvement in the aetiology of any disease in a tissue-specific manner prior the utilisation of a genetic knock out model and (c) the ability to investigate and distinguish the role of RIPK3 in inflammation independently of MLKL (mostly pro-apoptotic) from its role in MLKL-driven necroptosis.

In this study, we show how necroptosis can be molecularly detected in vivo. Following a thorough optimisation strategy, we provide a detailed methodology for the immunohistochemical and immunofluorescent in situ detection of S345-phosphorylated murine MLKL (pMLKL-S345) in different tissues (skin, intestine, lung, spleen and liver). We, first, optimise our detection method in genetic mouse models of necroptosis induction via conditional deletion of Caspase-8 (Casp8) or FADD from basal epidermal keratinocytes [34] or intestinal epithelial cells [35], respectively. Additionally, once demonstrated the validity and specificity of our protocol, we provide evidence of the applicability and advantages of detecting pMLKL-S345 in situ by utilising three additional models that uncover, clarify and support (1) the role of necroptosis in SARS-CoV infection, (2) the role of RIPK3 in inflammation independently of MLKL via the analysis of the chronic proliferative dermatitis mutation (cpdm) mice (*Sharpin*^*cpdm*/*cpdm*^) and *Sharpin*^*cpdm*/*cpdm*^; *Ripk3*^−/−^ animals and (3) the role of necroptosis in systemic inflammatory response syndrome (SIRS) following TNF injection. In some of these models we directly compare the in situ detection of pMLKL-S345 with the broadly used c-Casp3 demonstrating the importance of specifically detecting pMLKL-S345 in situ to identify which cells undergo necroptosis as well as highlighting functional and temporal differences with apoptotic cell death.

## Materials and methods

### Mouse models

The *Casp8*^*fl*/*fl*^ mice were kindly provided by Hedrick [36], *K14-Cre* [37], *Fadd*^*fl/fl*^ [38]*, Villin-Cre-ERT*^2^ [39] were obtained as previously described. *Shpn*^*m*/*m*^ mice were obtained from the Jackson laboratory [40]. *Mlkl*^*−/−*^ was generated by the Walczak lab as previously described [41], *Casp8*^*+/−*^ mice were kindly provided by Hakem [42], *Ripk3*^*−/−*^ animals by Dixit and Newton from Genentech [43]. C57Bl/6N wild-type mice were purchased from Charles River (Germany). Mice were bred to obtain *Casp8*^*fl/fl*^; *K14cre*^*tg/wt*^ carrying the conditional deletion of *Casp8* in keratinocytes (*Casp8*^*E-KO*^). Mice heterozygous for *Casp8* and homozygous for *Ripk3* (*Casp*^*+/−*^*; Ripk3*^*−/−*^) or *Mlkl* (*Casp8*^*+/*−^; *Mlkl*^*−/−*^) were bred to obtain *Casp8*^*−/−*^*; Ripk3*^−*/−*^ and *Casp8*^*−/−*^*; Mlkl*^−*/−*^ double-KO mice. To generate knock out mice with inducible intestinal-epithelial cell-specific FADD deletion (*Fadd*^*iIEC-KO*^), we crossed *Fadd*^*fl/fl*^ mice with *Villin-Cre-ERT*^2^ mice. These were further crossed with *Mlkl*^*−/−*^ to generate *Fadd*^*iIEC-KO*^; *Mlkl*^*−/−*^. *Fadd* deletion was induced by tamoxifen injections of 11 weeks old *Fadd*^*fl/fl*^*; Villin-Cre-ERT*^2^ and *Fadd*^*fl/fl*^*; Mlkl*^*−/−*^*; Villin-Cre-ERT*^2^ mice at 10 mg/mL (T5648, Sigma-Aldrich) for 3 consecutive days and then sacrificed at 14 weeks of age. As control, *Fadd*^*fl/fl*^ littermates not expressing Cre were treated with tamoxifen in the same way. All mice were kept in individually ventilated cages under a stable 12 h day/night cycle at the animal facilities of the Medical Faculty, the CECAD Research Center of the University of Cologne or the Medizinisch-Technisches Zentrum at the TU Dresden. Water and standard chow were provided ad libitum, room temperature and humidity were automatically maintained. Mice were daily monitored, scored blindly by two independent researchers and humanely sacrificed once reached endpoint criteria via cervical dislocation. Experiments were performed according to German animal protection laws and were approved by local ethics committees as well as local government authorities (*Landesamt für Natur, Umwelt und Verbraucherschutz Nordrhein* Westfalen or *Landesdirektion Dresden*, number: VSG2020.A022).

### SARS-CoV infection

Lung sections from SARS-CoV MA15-infected mice were obtained from a recently published study where experimental material and methods with regards to the model and infection are fully described [44]. SARS-CoV MA15 was kindly provided by S. Zúñiga and L. Enjuanes (CNB-CSIC, Madrid, Spain). Mice were infected intranasally with 10^6^ TCID50/ml in a total volume of 40 μl of PBS after isoflurane anaesthesia. A clinical score was meticulously derived, if deemed necessary, through the assessment of various components, including the appearance of the mouse, level of consciousness, activity level, responsiveness to stimuli, ocular appearance, and the frequency and quality of respiration. Mice were considered to have reached the humane endpoint when the clinical score was equal or exceeded 21, or when respiratory parameters surpassed a score of 3 [45], or when weight loss was >25% of the initial weight. Animals were kept under standard conditions of temperature, humidity and light at the Research Center on Encephalopathies and Emerging Diseases of the University of Zaragoza. Animal experimentation was approved by the Animal Experimentation Ethics Committee of the University of Zaragoza (number: PI44/20).

### Systemic inflammatory response syndrome (SIRS)

Co-housed 8–12 weeks old female C57Bl/6N mice were divided in pairs of two and each pair received two different doses of ice-cold recombinant, LPS-free mouse TNF (TNF) (575208, lot: B306769, BioLegend) in a final volume of 100 µl (dissolved in PBS) intravenously via tail-vein injection (i.v.). The administered doses were adjusted to 0.75 µg/g or 1 µg/g TNF. Control mice received a corresponding volume of ice-cold LPS-free PBS. The experiment was performed in a double-blinded manner. Mice were monitored every 2 h for body temperature drop and sacrificed 6 h after injection. Animal experimentation was approved by German animal protection laws and were approved by local ethics committees as well as local government authorities (number: TVA57/2017).

### Dermatitis scoring criteria

Skin lesions in *Shpn*^m/m^ mice were scored as previously described [40]. Briefly, two main clinical criteria were assessed to determine a total score: (1) the number of body regions presenting lesions and (2) the characteristics of each lesion. For the first criteria skin from four different body regions were assessed and appearance of lesions in each of these body regions generated a score of 1 (head = 1, neck = 1, back = 1 and flank = 1). For the second criteria, the characteristic of each lesion was defined by its severity. Punctuated small crusts received a score of 1, coalescent crusts received a score of 2 and ulceration received a score of 3. The sum of all individual scores according to these two was calculated to determine total severity. Scoring of skin lesions was performed by two independent researchers in a double-blinded fashion.

### Tissue harvesting and fixation

Mice were humanely sacrificed once reached endpoint criteria. Mouse skin samples were collected by harvesting skin biopsies from the dorsomedial region of the back of the mouse into a rectangle shape so that the long side of the rectangle follows the anterior–posterior axis of the mouse. Colon and small intestine were longitudinally cut and washed with PBS to remove faeces and then rolled up from proximal to distal (Swiss-rolls). Spleens, lungs and livers were carefully extracted, and washed in PBS. It was previously reported that crosslinking fixatives can interfere with specific detection of MLKL, RIPK1 and RIPK3 and methanol fixation was pointed as a more efficacious methodology to prevent false positive staining [26]. However, it is important to note that this conclusion was specific for detecting total and phosphorylated protein signal via immunofluorescence in adherent cells. In tissues, it is still a matter of debate if coagulant fixatives (ethanol and methanol) impact less on the steric configuration of the proteins and therefore on the antigenic site recognition [46, 47]. We opted for a formalin fixation because the advantages of formalin fixation include a good preservation of tissue morphology and because it is widely used for many different established antibodies, therefore, maximising the utilisation of the same tissue block (from the same animal) for the in situ detection of different markers. Importantly, formalin fixation sterilises tissues more reliably than precipitating fixatives, in particular for viruses (which is currently the requested fixative by European authorities for the transportation and utilisation of samples outside of S3 facilities such as SARS-CoV) and, therefore, relevant to investigate necroptosis activation following viral infection. In this study, we utilised tissues from different organs: skin, spleen, lung, gut and liver. Tissues were placed in an embedding cassette and fixed with either 10% buffered formalin (skin from *Casp8*^*E-KO*^, small intestine, colon and liver from TNF-induced shock model, HT501128-4L, Sigma-Aldrich) or 4% formaldehyde (skin and spleen from *Shpn*^*m/m*^ mice, lungs from the SARS-CoV infection model and small intestine and colon from *Fadd*^*iIEC-KO*^ mice, WAL60622, Walter CMP GmbH) overnight (O/N). The cassettes were stored in 70% EtOH (skin) or PBS (spleen, lung, small intestine, colon and liver) for at least 24 h before further processing.

### Tissue processing (paraffin embedding and microtomy)

Fixed tissues were paraffin embedded using a vacuum tissue processor (Model: ASP200S, Leica Biosystems) through standard automatic tissue embedding protocols. The paraffin-embedded blocks were cut to obtain 3 µm thick (lung, small intestine, colon and liver) or 5 µm thick (skin and spleen) consecutive sections, then placed on labelled polysine adhesion slide and dried O/N at 40 °C.

### Haematoxylin & eosin staining of paraffin-fixed tissues

Tissue sections were deparaffinised with xylene and rehydrated in reducing concentrations of EtOH (100%, 95%, 70%, 50 and 0%). The haematoxylin and eosin (H&E) staining was performed by incubating the tissue sections in haematoxylin (T865.3, Carl Roth) for 4 min, followed by thorough washing and a 15 min incubation in tap water. The slides were then incubated in eosin (X883.2, Carl Roth) for 30 s (skin) or 1 min (spleen, small intestine and colon), washed thoroughly in tap water, dehydrated (75%, 95%, 100% EtOH and xylene) and mounted (H-5700, Vector Laboratories).

### Optimisation strategy of in situ detection of pMLKL-S345 via IHC

The optimisation strategy took place at six different levels that we categorised as:

Level 1 – antibody selection, Level 2 – retrieval method, Level 3 – retrieval time, Level 4 – antibody dilution, Level 5 – usage of detergents, Level 6 – amplification method and in a total of 47 different conditions (Supplementary Fig. 1).

Out of all the commercially available antibodies claiming to detect pMLKL-S345 we decided to focus specifically on those that were known to provide a clean and reliable signal by western blotting and also those that had been most recently published in the literature to avoid any batch issues: a recombinant rabbit monoclonal antibody (EPR9515) to pMLKL-S345 antibody from abcam (ab196436) and a recombinant rabbit monoclonal antibody (D6E3G) to pMLKL-S345 antibody from Cell Signalling Technologies (CST, 37333) which was previously reported to successfully stain murine pMLKL-S345 in fixed adherent murine cells [26]. Importantly, the ab196436 was previously published to detect pMLKL-S345 in tissues via IHC [48]. However, despite several attempts, we (and others) could not obtain convincing results by following the published protocols, which therefore, prompted us to optmise the staining procedure.

It is well established that potential masking of epitopes arising from formalin fixation can be successfully overcome by the correct antigen retrieval. Initially, the two antibodies were tested with heat-induced epitope retrieval (HIER) method, in sodium citrate-based retrieval buffer, pH 6. Antibodies were diluted 1:200, 1:400, 1:800, 1:1600 and 1:2000. Standardised retrieval time for TintoRetriever Pressure Cooker (10 min) and a previously verified maximum retrieval time (13 min) were compared. In addition, an avidin–biotin–HRP signal amplification system was implemented to improve signal detection (data with regular anti-rabbit HRP conjugated antibody not shown). Under these conditions, the abcam antibody failed to show signal specificity because of high background levels and consequent low signal-to-noise ratio. The CST antibody showed promising results at this initial stage and was further validated. To improve antigen retrieval, a commercially available Tris-EDTA based buffer, pH 9 (S236784-2, Agilent Technologies) was utilised, as Tris-EDTA based buffers at pH 9 are usually employed for either low affinity antibodies or when difficult epitopes require more effective retrieval. Different retrieval times were tested: 5, 7 (data not shown), 10 and 13 min. In parallel, an enzymatic/proteolytic antigen retrieval method utilising Proteinase K (20 µg/ml in TE Buffer: 50 mM Tris Base, 1 mM EDTA, 0.5% Triton X-100, pH 8.0) was tested. From this, we concluded that pressure cooker-based HIER using Tris-EDTA based buffer, pH 9 and 1:2000 antibody dilution of the pMLKL-S345 antibody from Cell Signalling Technologies (CST, 37333) were the most favourable conditions to detect in situ pMLKL-S345. To further optimise the signal/noise ratio, 0.05% v/v Tween20 was added to the retrieval buffer to obtain better permeabilisation. Serial antibody dilutions (CST37333) of 1:2000–1:10000 were further assessed to test background detection. Since further dilutions above 1:2000 incurred into decreased antibody-signal detection, despite decreasing background, we decided to carry forward with 1:2000 dilution of the CST37333 antibody. We also validated the staining method with a ‘homemade’ 0.05% v/v Tween20, Tris-EDTA based retrieval buffer, pH 9 and HIER using a standard, commercially available microwave oven.

### Immunostaining

After standard deparaffinisation and rehydration, the paraffin sections were retrieved differently for the detection of different antibodies. For pMLKL-S345 detection (37333, Cell Signalling Technology, 1:2000) slides were retrieved in 0.05% v/v Tween20, Tris-EDTA based antigen retrieval buffer, pH 9 (S236784-2, Agilent Technologies) for 13 min at 114–121 °C using the TintoRetriever Pressure Cooker (BSB7008, BioSB). For c-Casp3 staining (9664, Cell Signalling Technology, 1:100, for skin sections and 9661, Cell Signalling Technology, 1:200, for spleen, lung, small intestine, colon and liver) slides were retrieved in sodium citrate buffer, pH 6 (C9999, Sigma-Aldrich) for 10 min at 114–121 °C using the TintoRetriever Pressure Cooker. Endogenous peroxidase activity, antibody non-specific binding sites, avidin binding sites and endogenous biotin and biotin receptors were blocked according to the manufacturer’s instructions (SP-6000-100, SP-5035-100 and SP-2001, Vector Laboratories, respectively). Biotinylated goat anti-rabbit secondary antibody (1:200, BA-1000, Vector Laboratories) in combination with either the avidin–biotin–HRP amplification system (PK-6100, Vector Laboratories) with DAB substrate (SK-4105, Vector Laboratories) or Alexa Fluor 594 Streptavidin (S11227, Thermo Fisher Scientific) were used for antibody detection. All sections were counterstained with haematoxylin (H-3401, Vector Laboratories) or DAPI (D9564, Sigma-Aldrich). The sections were then mounted using commercially available mounting media (H-5700, Vector Laboratories or P36930, Thermo Fisher Scientific).

### Lambda protein phosphatase (lambda PP) treatment

Slides were treated with lambda PP (40.000U/ml) diluted in Lambda PP buffer (P0753S, New England Biolabs) or with Lambda PP buffer alone according to manufacturer instructions. Slides were incubated at 37 °C for 24 h in a wet chamber and then washed three times (5 min) in TBS-T 0.1% v/v Tween20 before being incubated with the pMLKL-S345 antibody as described above.

### Histopathological analysis

Histopathological evaluation was performed in H&E stained 3 µm thick intestinal Swiss-roll sections using the scoring system as described before [35]. Briefly, histopathology scores are composed of four parameters: epithelial hyperplasia, epithelial injury, tissue inflammation and epithelial cell death. Histological sub-scores for each parameter: 0, absent; 1, mild; 2, moderate; 3, severe. An ‘area factor’ for the fraction of affected tissue was assigned and multiplied with the respective parameter score (1 = 0–25%; 2 = 25–50%; 3 = 50–75%; 4 = 75–100%). Each area was scored individually and multiplied with the correlating area factor. Total histology score was calculated as a sum of all parameter scores multiplied with their area factors. Maximum score was 48. Evaluation was performed in a blinded fashion.

### Imaging

Immunohistochemistry slides were digitalised via acquisition in a digital slide scanner (NanoZoomer S360MD Slide scanner system, Hamamatsu Photonics K.K.) with 40× magnification lens. Additional bright field images were acquired with Leica DM750 Binocular microscope and LAS X Life Science Microscope Software, Leica, objective 10×, 20× or 63×. Immunofluorescence pictures were acquired with LSM 980 with Airyscan 2 and ZEN Microscopy Software, Zeiss, objective 20× and 63×, Zeiss.

### Image analysis

The acquired bright field images were analysed in QuPath, an open-source software [49]. To quantify the positively stained cells, a pixel classifier was generated in order to exclude tissue areas that should not be accounted for during the quantification step and would otherwise interfere with the ‘positive cell detector’ leading to false positive results. After setting the parameters and threshold, the ‘positive cell detector’ was run and the quantification results were acquired. The percentages of positive cells per total number of cells for each section or image for the different biological replicates were plotted utilising GraphPad. One-way or two-way Anova statistical analysis and Tukey’s multiple comparisons test or unpaired two-tailed *t*-test was calculated via graph pad/prism to determine significance among the different groups (*P ≤ 0.05, **P ≤ 0.01, ***P ≤ 0.001, ****P ≤ 0.0001, ns = not significant).

## Results

### Reliable in situ detection of pMLKL-S345 allows to distinguish apoptotic from necroptotic cell death in different subcellular populations

**Fig. 1:**
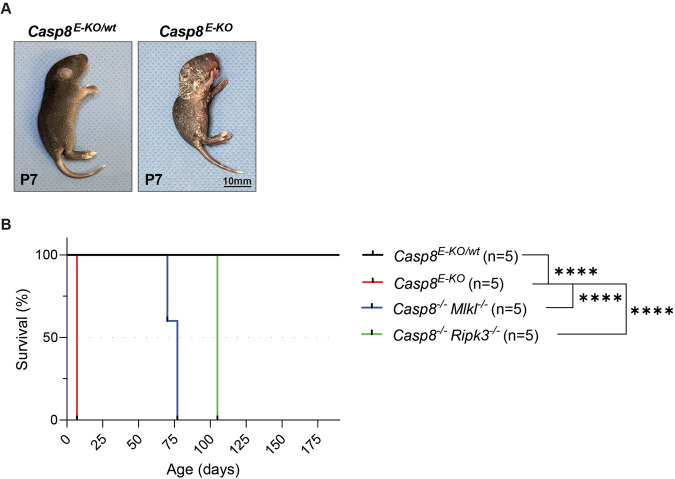
*Casp8*^*E-KO*^ is a reliable and robust model to study necroptosis. **A** Representative images of *Casp8*^*E-KO*^ mice at the indicated time point (P7). Control mice included littermates with the *Casp8*^*E-KO/wt*^ genotype. **B** Kaplan–Meier survival graph of mice of the indicated genotypes (*n* = 5 in each group). *P* values were calculated by Gehan–Breslow–Wilcoxon test. ****P ≤ 0.0001.

To initially optimise and validate the in situ detection of pMLKL-S345 we decided to employ a published model of Casp8 deletion from epidermal keratinocytes (*Casp8*^*E-KO*^). These mice are known to succumb to aberrant skin inflammation induced by RIPK3 and MLKL-mediated necroptosis. Accordingly, mice bearing deletion of Casp8 from epidermal keratinocytes (*Casp8*^*E-KO*^) presented with severe skin inflammation leading to lethality by P7 [34] (Fig. 1A, B). Mice with heterozygous deletion of Casp8 from keratinocytes (*Casp8*^*E-KO/wt*^*)* showed no sign of inflammation, neither at P7 nor at a later stage throughout their monitored life span and were indistinguishable from wild-type mice (Fig. 1A, B). These mice were utilised as negative controls to validate the pMLKL-S345 antibody and staining specificity in the presence of the single K14-*Cre* allele. As previously reported, the embryonic lethality induced by constitutive Casp8 deficiency is completely rescued by genetic removal of either RIPK3 or MLKL suggesting that its pathological cause is due to aberrant necroptosis [34]. Since removal of MLKL or RIPK3, the kinase responsible for phosphorylation of MLKL at S345, would impede detection of pMLKL-S345, we utilised *Casp8*^*−/−*^*; Mlkl*^−*/−*^ and *Casp8*^*−/−*^*; Ripk3*^*−/*−^ mice as further negative controls. Consistent with the absence of necroptosis and with the notion that necroptosis induction is responsible for the lethal dermatitis, these mice presented no sign of necroptosis-induced skin inflammation and their survival was significantly prolonged (Fig. 1B). Due to the splenomegaly and lymphadenopathy that occurs in *Casp8*^*−/−*^*; Mlkl*^−*/*−^ and *Casp8*^*−/−*^*; Ripk3*^−*/−*^ mice, they were sacrificed once endpoint criteria were reached (Fig. 1B) and skin samples were collected for immunohistochemical analysis.

**Fig. 2:**
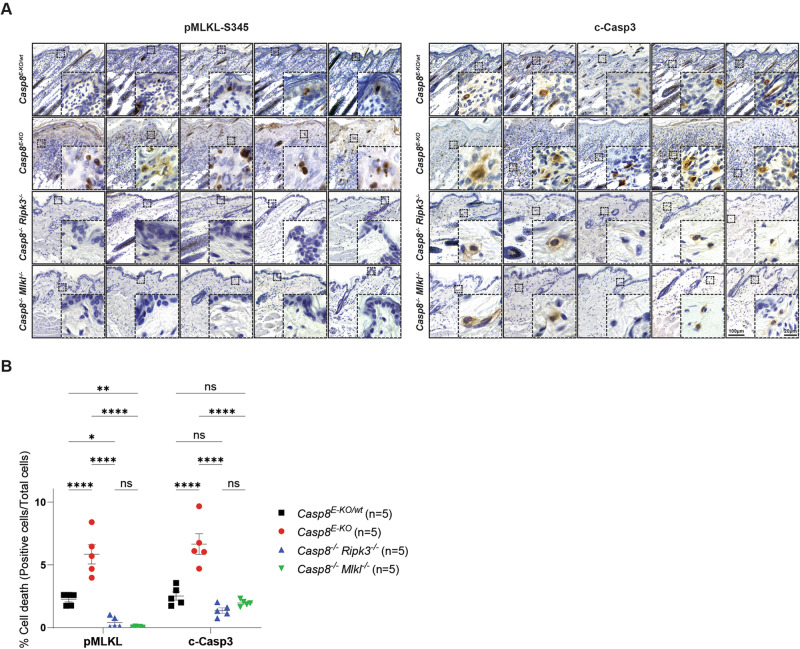
Reliable detection of pMLKL-S345 in situ distinguishes necroptosis from apoptosis. **A** Representative bright field images of skin sections from mice with the indicated genotypes immunostained with pMLKL-S345 and c-Casp3. Consecutive skin sections from the same mice were utilised for each marker and are shown in the same order (*n* = 5 in each group). Scale bars: 100 µm (representative field) and 20 µm (magnified selected area). **B** Graph showing quantification of each immunostaining obtained via QuPath after slides were digitalised in a digital slide scanner as described in the Supplementary Methods section. For each marker, total skin section was analysed and total numbers of cells were obtained to calculate the percentage of positive cells over the total amount of cells detected. Data are presented as mean + SEM and each dot represents one mouse. *P* values were calculated via two-way Anova. *P ≤ 0.05, **P ≤ 0.01, ****P ≤ 0.0001, ns not significant.

After establishing our model of choice, we selected two pMLKL-S345 antibodies: a previously published recombinant rabbit monoclonal antibody (EPR9515) to pMLKL-S345 antibody from abcam [48, 50] and a recombinant rabbit monoclonal antibody (D6E3G) to pMLKL-S345 antibody from Cell Signalling Technologies (CST, 37333) which we tested on skin sections obtained from the *Casp8*^*E-KO*^ animals. After performing different optimisation conditions (47 steps shown in Supplementary Fig. 1), we concluded that optimal detection of pMLKL-S345 takes place using antibody 37333 (CST) with a 1:2000 dilution using 0.05% v/v Tween20, Tris-EDTA based antigen retrieval buffer, pH 9 (S236784-2, Agilent Technologies), retrieving for 13 min at 114–121 °C. Details of the optimisation steps are provided in the ‘Materials and Methods’ section and in Supplementary Materials and Methods (Supplementary Fig. 1). Once the optimal staining conditions were determined, consecutive skin sections from five mice of each of the above-mentioned genotypes were stained for pMLKL-S345 or c-Casp3 and counterstained with haematoxylin to visualise tissue architecture as well as to distinguish different subcellular populations (Fig. 2A). Dying keratinocytes in the epidermis of *Casp8*^*E-KO*^ mice showed clear and specific staining for pMLKL-S345 demonstrating activation of the necroptosis executioner MLKL (Fig. 2A, B). Significantly lower signal was detected in the *Casp8*^*E-KO/wt*^ mice whereas no staining was detectable in *Casp8*^*−/−*^*; Mlkl*^*−/−*^ or *Casp8*^*−/−*^*; Ripk3*^*−/−*^ mice (Fig. 2A, B). Similarly, c-Casp3 was also detected in *Casp8*^*E-KO*^ mice, however, in contrast to pMLKL-S345, c-Casp3-positive cells, specifically fibroblasts and immune cells, were mostly located in the dermis (Fig. 2A). Although the absence of pMLKL-S345-positive cells in *Casp8*^*−/−*^*; Ripk3*^−*/−*^ would already exclude the possibility that the selected antibody would detect non-specifically total MLKL, we also decided to pre-treat skin samples obtained from *Casp8*^*E-KO*^ with lambda PP to remove phosphate groups from phosphorylated residues to further validate the specificity of our protocol with the antibody of choice. Lambda PP treatment completely removed any pMLKL-S345 signal detected from consecutive skin sections obtained from *Casp8*^*E-KO*^ animals (Supplementary Fig. 2), further confirming specificity of detection of the phosphorylated target.

These results demonstrate that the described protocol serves to specifically stain pMLKL-S345 in keratinocytes undergoing necroptotic cell death in the epidermis. Importantly, the staining pattern obtained thereby was clearly distinct from that obtained when staining for c-Casp3 in consecutive tissue sections. The latter staining identified other cells that underwent apoptotic cell death in the dermis, likely as a consequence of the necroptotic death of keratinocytes in the epidermis, as c-Casp3-positive cells were largely absent from the different control tissues (Fig. 2A, B).

### In situ detection of pMLKL-S345 is applicable to immunofluorescence staining

**Fig. 3:**
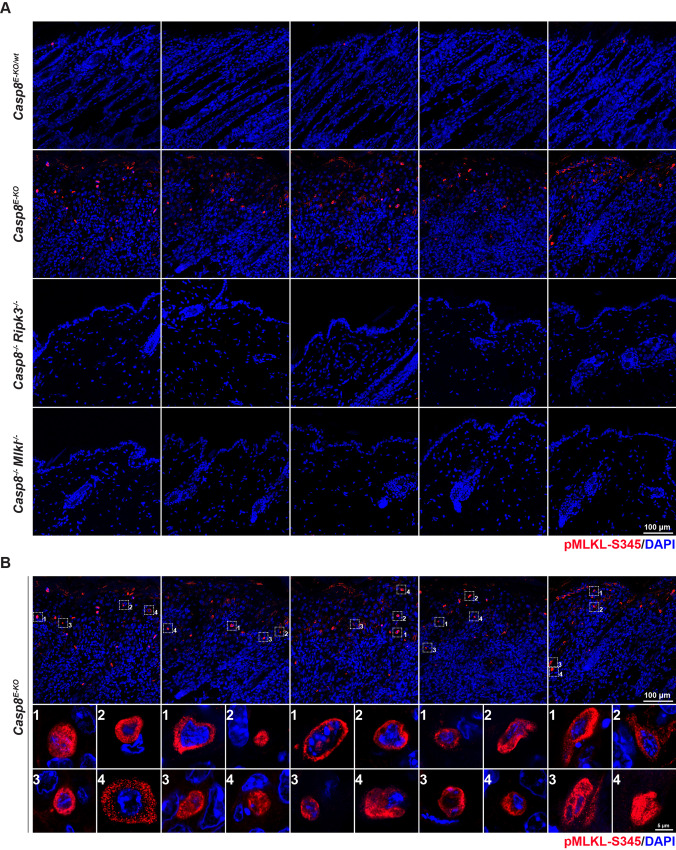
In situ detection of pMLKL-S345 is applicable to immunofluorescence staining. **A**, **B** Representative images of skin sections from mice with the indicated genotypes immunostained with Alexa fluor 594 streptavidin (red) to detect pMLKL-S345 and with DAPI (blue) to detect nuclei (*n* = 5 in each group). Dashed squares indicate 4 representative pMLKL-S345-positive cells per image. Scale bars: 100 µm. **B** Representative images (single slice 0.4 μm) of pMLKL-S345 positive cells (*n* = 4 cells per mouse in a total of *n* = 5 mice). Scale bar: 5 µm.

Having established a specific and sensitive protocol for the in situ detection of pMLKL-S345 in skin sections via immunohistochemistry, we next determined whether this protocol also served to detect pMLKL-S345 by immunofluorescence via confocal microscopy in skin and intestinal sections. To this end, we applied slight modifications to the protocol due to the nature of the different fluorophore-attached secondary antibodies required for acquisition via laser excitation (details provided in ‘Materials and Methods’ and in Supplementary Material and Methods). Comparably to the immunohistochemical detection of the above-mentioned samples (Fig. 2B), our data showed specific staining of cells in the skin sections obtained from *Casp8*^*E-KO*^ mice while samples from both *Casp8*^*−/−*^*; Mlkl*^−*/−*^ or *Casp8*^*−/−*^*; Ripk3*^−*/−*^ showed a clear lack of any detectable signals (Fig. 3A, B). Additionally, we employed the conditional deletion of *Fadd* in the intestinal epithelium via tamoxifen injection previously shown to lead to the disruption of the intestinal barrier via activation of necroptosis [35]. Briefly, *Fadd*^*fl/fl*^ mice were crossed with *Villin-Cre-ERT*^2^ to obtain *Fadd*^*iIEC-KO*^. These were further crossed with the *Mlkl*^*−/−*^ to generate the *Fadd*^*iIEC-KO*^*; Mlkl*^*−/−*^ which served as a negative control. At 11 weeks of age, these mice were injected with 1 mg tamoxifen (10 mg/ml) for 3 consecutive days to obtain the deletion of FADD from intestinal epithelial cells (*Fadd*^*iIEC-KO*^ and *Fadd*^*iIEC-KO*^; *Mlkl*^*−/−*^). Age-matched control littermates (*Fadd*^*fl/fl*^) were also injected with tamoxifen and served as experimental controls (*Fadd*^*IEC-WT*^). Mice were sacrificed 3 weeks after the first tamoxifen injection (14 weeks) and intestinal samples were analysed for pMLKL-S345 detection by confocal microscopy. The ileum, duodenum and jejunum obtained from *Fadd*^*iIEC-KO*^ animals showed intestinal epithelial cells positive for pMLKL-S345 with the ileum presenting the greatest number of positive cells and duodenum, jejunum and colon presenting progressively less to no positive cells for pMLKL-S345 (Supplementary Fig. 3). There was no detectable staining of pMLKL-S345 in any intestinal sample obtained from control *Fadd*^*IEC-WT*^ or from *Fadd*^*iIEC-KO*^; *Mlkl*^*−/−*^. Conclusively, these data show the ability to specifically detect pMLKL-S345 via immunofluorescence and subsequent confocal microscopy acquisition, similarly to the detection shown via immunohistochemistry. Moreover, we highlight the usefulness of this staining in further detecting MLKL-driven necroptosis in different areas of the intestine, granting the possibility to precisely determine the exact site and extent of necroptotic cell death-induced inflammation.

### SARS-CoV infection induces apoptotic and necroptotic cell death

**Fig. 4:**
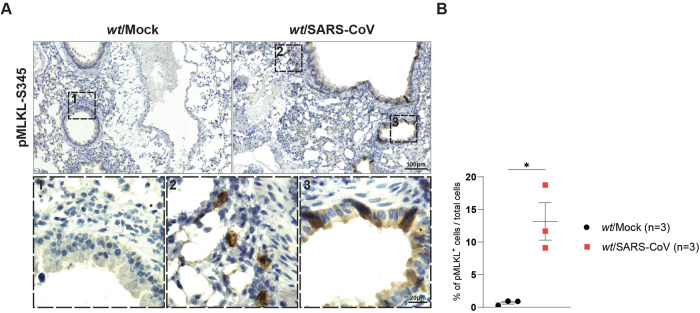
SARS-CoV infection induces apoptotic and necroptotic cell death. **A** Representative bright field images of lung sections of wt mice infected intranasally with 10^6^ TCID50/ml SARS-CoV MA15 virus at 3 days post-infection (dpi) immunostained with pMLKL-S345 (*n* = 3 for mock-infected and MA15-infected mice). Scale bars: 100 µm (representative field) and 50 µm (magnified selected area). **B** Graph showing quantification of pMLKL-S345 immunostaining obtained via QuPath after slides were digitalised in a digital slide scanner as described in the Supplementary Methods section. Total lung sections were analysed and total numbers of cells were obtained to calculate the percentage of positive cells over the total amount of cells detected. Data are presented as mean + SEM and each dot represents one mouse. *P* values were calculated via one-way Anova, Tukey’s multiple comparisons test. *P ≤ 0.05.

Lagunas et al. recently showed that infection by mouse adapted SARS-CoV MA15 (SARS-CoV) leads to cell death in the bronchioles and alveoli of infected mice [44]. In this study, while apoptosis was shown to play a role (via detection of c-Casp3-positive cells) it was clear that other modalities of cell death are engaged following SARS-CoV infection as a greater number of TUNEL-positive cells, although c-Casp3-negative, were detected in sections obtained from the lungs of infected mice [44]. To test the involvement of necroptosis activation, we obtained consecutive lung sections from the published mock-infected and SARS-CoV-infected mice [44] and performed pMLKL-S345 staining in situ on these sections. Our data clearly show a significant amount of pMLKL-S345-positive staining in alveolar cells as well as in cells of different origin in the lung epithelium, demonstrating activation of necroptosis following infection (Fig. 4). Considering the previously reported data, it is plausible that both apoptosis and necroptosis contribute to the inflammatory syndrome observed in these animals following SARS-CoV infection and that the inhibition of both could be a valuable therapeutic strategy. These data show the importance of pMLKL-S345 staining to investigate and determine the occurrence of necroptosis during infection allowing to distinguish and understand how different cell death modalities affect pathological and systemic inflammation following viral infection.

### pMLKL-S345 staining distinguishes between RIPK3-mediated necroptosis and RIPK3-mediated inflammation independently of necroptosis

**Fig. 5:**
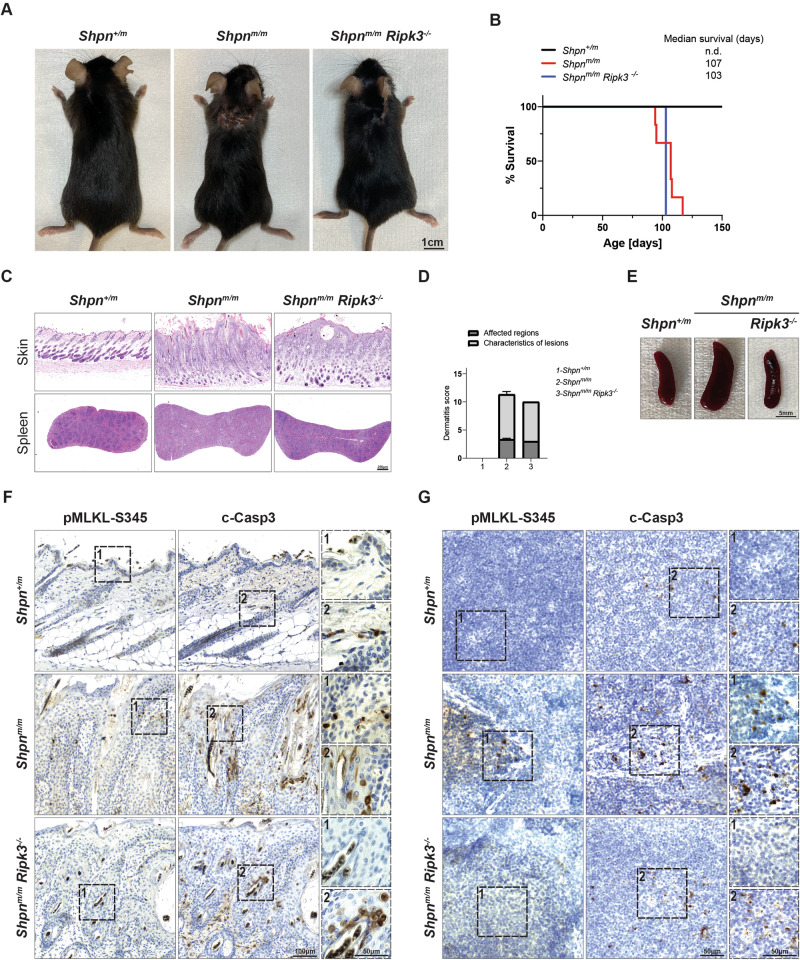
pMLKL-S345 staining distinguishes between RIPK3-mediated necroptosis and RIPK3-mediated inflammation. **A** Representative images of mice of indicated genotypes at the endpoint (14–15 weeks). Control mice included littermates with the *Shpn*^*+/m*^ genotype. **B** Kaplan–Meier survival graph of mice with the indicated genotypes (*n* = 7 for *Shpn*^*+/m*^, 6 for *Shpn*^*m/m*^, 1 for *Shpn*^*m/m*^*; Ripk3*^*−/−*^ mice). **C** Representative images of skin and spleen sections from mice with the indicated genotypes stained with H&E. Slides were digitalised in a digital slide scanner and images were acquired in QuPath. **D** Graph of severity score of dermatitis assessment in 14–15 weeks old mice of the indicated genotypes. *Shpn*^*+/m*^ mice (14–21 weeks old) served as controls (*n* = 5 for *Shpn*^*+/m*^, 6 for *Shpn*^*m/m*^, 1 for *Shpn*^*m/m*^*; Ripk3*^−*/*−^ mice). **E** Representative bright field images of spleens from mice of the indicated genotypes. **F**, **G** Representative bright field images of skin (**F**) and spleen (**G**) sections from mice with the indicated genotypes immunostained with pMLKL-S345 and c-Casp3. Consecutive skin sections from the same mice were utilised for each marker and displayed according to their order (*n* = 3 for *Shpn*^*+/m*^, 3 for *Shpn*^*m/m*^ and 1 for *Shpn*^*m/m*^*; Ripk3*^*−/−*^ mice). Scale bars: skin (**F**) 100 µm (representative field) and 50 µm (magnified selected area), spleen (**G**) 50 µm for both representative field and magnified selected area.

cpdm mice bear the genetic deficiency of *Sharpin* (*Sharpin*^*cpdm/cpdm*^, henceforth referred as *Shpn*^*m/m*^) [51–54]. These animals are characterised by dermatitis and multi-organ inflammation including splenomegaly, liver inflammation, disrupted lymphoid architecture and loss of Peyer’s patches in the gut. Overall, TNF/TNFR1-induced cell death has been demonstrated to drive these phenotypes [51, 53, 54]. Specifically, TNF-induced apoptosis is responsible for skin inflammation since *Casp8* heterozygosity can significantly ameliorate the dermatitis, while multi-organ inflammation is predominantly driven by RIPK3/MLKL-induced necroptosis [53, 54]. Interestingly, while co-deletion of Sharpin and RIPK3 delays the onset of the dermatitis, MLKL co-deletion does not affect the skin inflammation observed in the *Shpn*^*m*/*m*^ [53, 54]. Previous studies that have addressed this issue have inferred that attenuated linear ubiquitination unleashes a RIPK3 function which is inflammatory in the skin, yet independent of MLKL. In other tissues (e.g., the spleen), RIPK3 exerts a purely necroptotic function [53, 54]. To further validate these conclusions via the in situ detection of pMLKL-S345, we generated the *Shpn*^*+*/*m*^, *Shpn*^*m*/*m*^ and *Shpn*^*m*/*m*^; *Ripk3*^−*/*−^ mice. Consistent with previously published reports, *Shpn*^*m*/*m*^ mice manifested onset of dermatitis at around 11 weeks of age while *Shpn*^*m*/*m*^; *Ripk3*^−*/−*^ animals presented a modest delay in the appearance of the dermatitis [53, 54]. Nevertheless, they were all sacrificed at 14–15 weeks of age after reaching endpoint criteria (Fig. 5A, B). In accordance with previous studies [53, 54] histological analysis showed a very mild amelioration of the epidermal thickness in *Shpn*^*m*/*m*^; *Ripk3*^−*/−*^ which was confirmed by the dermatitis score (Fig. 5C, D). As expected *Shpn*^*m*/*m*^; *Ripk3*^−/−^ mice presented a clear restoration of the marginal zone in the spleen which was reflected by a complete rescue of the splenomegaly observed in the *Shpn*^*m*/*m*^ mice (Fig. 5C, E). We then obtained consecutive skin and spleen sections and compared the staining of pMLKL-S345 and c-Casp3 in situ. There was a very minor increase in the levels of pMLKL-S345 detected in localised areas of the skin of *Shpn*^*m*/*m*^ compared to *Shpn*^*+*/*m*^ mice, suggesting that MLKL-induced necroptosis contributes marginally to the dermatitis observed in the *Shpn*^*m/m*^ animals. In contrast c-Casp3 staining was distinctly prominent in the skin of *Shpn*^*m*/*m*^ mice as compared to *Shpn*^*+*/*m*^ mice and only partially diminished in *Shpn*^*m*/*m*^*; Ripk3*^*−/−*^ animals, as previously published. This is suggesting that RIPK3-mediated inflammation contributes to dermatitis independently of MLKL activation and that apoptotic cell death is the main driver of dermatitis. It is tempting to speculate that apoptosis can, in turn, cause necroptosis to such a low extent that it does not contribute to the lethality of the phenotype. On the other hand, detection of pMLKL-S345 revealed a clear necroptotic phenotype in the spleen of the *Shpn*^*m*/*m*^ which was completely absent in the spleen of *Shpn*^*m*/*m*^*; Ripk3*^*−/−*^ animals. These data suggest that, attenuation of linear ubiquitination, differently from TNF-induced apoptosis in the skin, leads to RIPK3/MLKL-mediated necroptosis and secondary apoptosis in the spleen.

### Systemic inflammatory response syndrome drives apoptosis and not necroptosis in intestinal epithelial cells

**Fig. 6:**
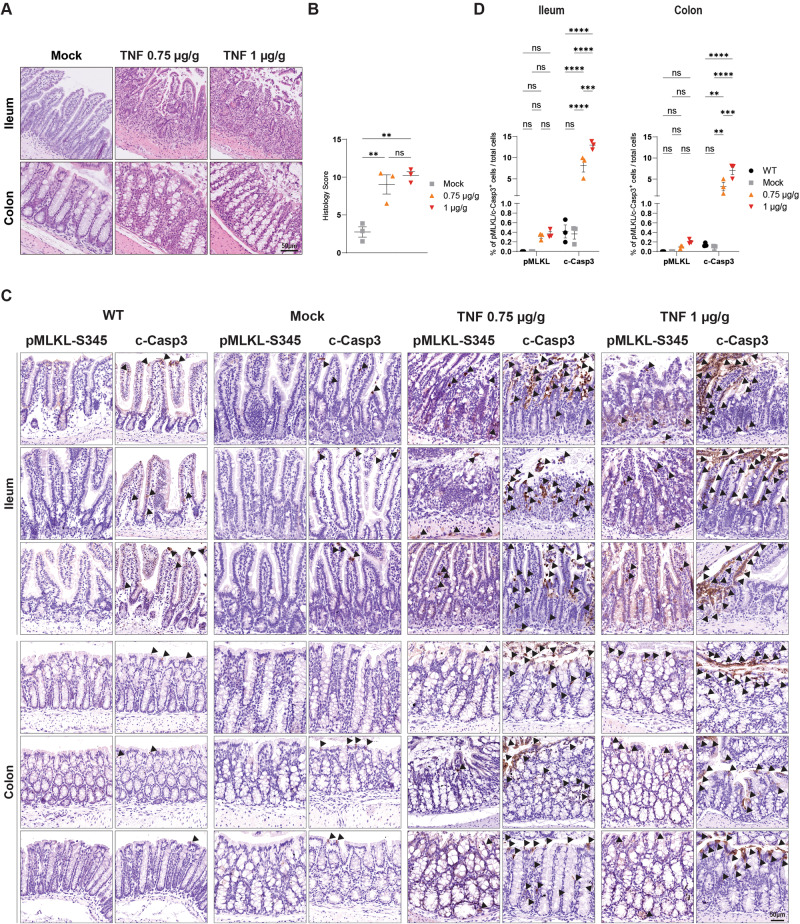
Systemic inflammatory response syndrome drives apoptosis and not necroptosis in intestinal epithelial cells. Co-housed 10–12 week old female C57Bl6/N wt mice were injected with 0.75 µg/g or 1 µg/g of ice-cold recombinant, LPS-free mouse TNF intravenously. Control mice received a corresponding volume of ice-cold LPS-free PBS. Mice were sacrificed 6 h post-injection. Untreated wt mice were additionally analysed (*n* = 3 in each group). **A** Representative images of small intestine and colon sections (Swiss-rolls) of wt mice as indicated were stained with H&E. Slides were digitalised in a digital slide scanner and pictures were acquired in QuPath. Scale bars: 50 µm. **B** Graph showing histology score in whole intestine. Data are presented as mean + SEM and each dot represents one mouse. *P* values were calculated via one-way Anova, Tukey’s multiple comparisons test. **P ≤ 0.01, ns not significant. **C** Representative images of ileum and colon sections (Swiss-rolls) of wt mice as indicated immunostained with pMLKL-S345 and c-Casp3. Slides were digitalised in a digital slide scanner and pictures were acquired in QuPath. Scale bars: 50 µm. Arrowheads indicate pMLKL-S345-positive cells and c-Casp3-positive areas. **D** Graph showing quantification of illustrated pictures for each immunostaining obtained via QuPath after slides were digitalised in a digital slide scanner as described in the Supplementary Methods section. Total numbers of cells were obtained to calculate the percentage of positive cells over the total amount of cells detected. Data are presented as mean + SEM and each dot represents one mouse. *P* values were calculated via two-way Anova, Tukey’s multiple comparisons test. **P ≤ 0.01, ***P ≤ 0.001, ****P ≤ 0.0001, ns not significant.

It was previously shown that in the C57BI/6 N background, *Casp8*^*−/−*^*; Mlkl*^−*/−*^ mice are completely unresponsive to TNF-induced SIRS [55]. While SIRS, as determined by body hypothermia, serum cytokines and chemokines levels and SIRS-induced mortality is significantly ameliorated in both RIPK3-deficient or RIPK1-catalytically inactive mice, *Mlkl*^−*/−*^ animals have shown different responses to high compared to low doses of TNF [55]. Specifically, while *Mlkl*^−*/−*^ mice seem to respond similarly to WT animals when treated with low doses of TNF, differences with *Ripk3*^−/−^ are less evident following injections of high doses of TNF [55]. This puts into questions whether the unresponsiveness of the *Casp8*^*−/−*^*; Mlkl*^−*/*−^ mice to SIRS (C57BI/6N) is solely due to loss of apoptosis or both, apoptosis and necroptosis and, most importantly, whether RIPK3 might play a role in inflammation independently of MLKL also in this pathological context. Considering that homozygous deletion of Casp8 leads to embryonic lethality [56], it is impossible to determine whether MLKL deletion is simply required to prevent aberrant necroptosis (as a consequence of Casp8 deletion) or whether MLKL-induced necroptosis is indeed involved in SIRS. To answer this question, we injected female C57BI/6N mice (i.e., the same strain as in the study reported above [55]) with low or high doses of TNF (via tail-vein injection) and sacrificed them after 6 h for tissue harvesting and subsequent intestinal IHC staining with pMLKL-S345 and c-Casp3 (Fig. 6A–D). Histological analysis confirmed the intestinal damage as a result of TNF injections (Fig. 6A, B). Our data conclusively show that both doses of TNF drive predominantly apoptosis and not necroptosis, as in the area of intestinal damage we could only detect a significant increase of c-Casp3-positive cells (Fig. 6C, D and Supplementary Fig. 4). Specifically, different parts of the gastrointestinal tract, including ileum, duodenum, jejunum and colon, contained cells positive for c-Casp3 staining, with ileum and duodenum showing the highest levels. It was previously reported that necroptosis-induced liver damage contributes to TNF-mediated mortality in C57BI/6J animals [57]. Unexpectedly, we could not detect a high number of pMLKL-S345-positive cells (although significant) nor a difference in c-Casp3 staining compared to control in our C57BI/6N animals (Supplementary Fig. 5). Together, these results suggest that, at least in the C57BI/6N background, the previously published amelioration observed in RIPK3-deficient mice might be due to the ability of RIPK3 to promote apoptosis and inflammation independently of necroptosis execution. This notion is further supported by the published improvement observed in *Casp8*^+/−^ animals or in RIPK1-kinase-dead mice (*Ripk1*^*K45A*^) of the same background [55]. On the other hand, the amelioration observed upon MLKL or RIPK3 deficiency following injection of high doses of TNF might be the result of secondary systemic necroptosis as a consequence of apoptosis execution which leads to the disruption of the intestinal epithelial barrier.

## Discussion

Currently, the detection of c-Casp3 is widely employed as a tissue marker of cell death, and whilst it has been shown to detect also Casp8-induced pyroptosis [35], it has paradoxically been utilised even in models in which the deletion of FADD or Casp8 and RIPK1 leads to necroptosis-induced lethality which has been genetically demonstrated to occur in a caspase-independent manner as completely rescued by RIPK3 co-deletion [58–60]. Hence, since necroptosis execution is not dependent on the cleavage of caspases, it follows that the cleavage of Casp3 cannot be used to specifically identify cells that undergo necroptotic cell death but rather cell death events that take place as a consequence of it. In the present study, we developed a protocol for the specific immunohistochemical and immunofluorescence staining of pMLKL-S345 in tissue specimens (skin, intestine, lung, spleen and liver) obtained from different animal models. While we acknowledge the difficulty to detect a cell death marker in every tissue, we believe that this protocol can be further extended to other tissues provided a certain degree of tissue-specific optimisation of which we provide ample example. In particular in the kidney where specifically the abcam antibody (compared in this study and excluded) has been used to successfully stain for pMLKL-S345 [50], however, deemed unspecific in another study [61].

Here, we demonstrate significant advantages of this staining over others previously employed in characterising the aetiology of different cell death-driven or involving pathologies, potentially substituting the need for genetic crosses, and at the same time, providing more informed conclusions on the role of pMLKL-S345-induced necroptosis. The comparison of the immunohistochemical staining we obtained with the in situ detection of pMLKL-S345 via the newly developed protocol to the ones obtained by staining c-Casp3 in the *Casp8*^*E-KO*^ model, critically highlights important differences between these two markers of cell death. Despite the clear presence of pMLKL-S345 in the epidermis containing dying keratinocytes, the dermal layer presented a significant number of cells that stained positive for c-Casp3 which, histologically, correspond to fibroblasts and immune cells. Since the subcellular population positive for each marker was clearly different as well as located in distinct layers of the skin, we concluded that c-Casp3 staining detected a secondary cell death event in cells in which Casp8 is expressed. It is in fact known that necroptotic cell death, via the release of the content of the dying cells, which includes TNF, type I interferon, IL-6, IL-33, IL-36, HMGB1, IL-1α [62, 63], initiates a wave of systemic inflammation further augmented by other modalities of cell death including apoptosis, GSDME-mediated mitochondria pore formation and Casp8-induced pyroptosis [16, 25, 28, 35, 64, 65]. Furthermore, release of inflammatory cytokines also drives the recruitment of immune cells expressing other death ligands such as TRAIL and FasL, in addition to TNF [66], likely responsible for the cell death observed in the dermis in both fibroblasts and immune cells in the present study.

We then further extended the in situ detection of pMLKL-S345 to *Fadd*^*iIEC-KO*^ allowing, for the first time the assessment of different degrees of necroptosis in different areas of the intestine. *Fadd*^*IEC-KO*^ were previously shown to suffer from severe intestinal inflammation [35]. While TNF or TNFR1 co-deletion together with co-deletion of ZBP1, significantly ameliorated colon inflammation, by preventing MLKL-induced necroptosis, this is not sufficient to prevent ileitis, where it was proposed that Casp8 also drives Gasdermin-D-mediated pyroptosis of FADD-deficient intestinal epithelial cells which contributes to the observed inflammatory phenotype [35, 67]. Importantly, MLKL co-deletion can completely prevent colitis while ileitis is only completely prevented by co-deletion of MLKL in *Fadd*^*IEC-KO*^; *Casp8*^*IEC-KO*^. In all published studies both colitis and ileitis were characterised by histological score, CD45 and c-Casp3 staining [35, 67, 68]. Specifically, the latter was employed only to assess residual Casp8 activity in the *Fadd*^*IEC-KO*^; *Casp8*^*IEC-KO*^ mice in the small intestinal epithelium and CD45 was used as the marker of choice to detect intestinal inflammation. Our pMLKL-S345 detection protocol allows, for the first time, direct detection of necroptotic cell death in situ, confirming the prominent occurrence of necroptosis in the ileum. Surprisingly, we could not detect pMLKL-S345 in the colon probably due to either the early time point at which the tamoxifen-treated mice were sacrificed or potentially highlighting substantial differences between embryonic conditional deletion of FADD versus adult conditional deletion of FADD in the intestinal epithelium.

While many viruses were shown to trigger both Casp8-mediated apoptosis and RIPK1/RIPK3- or ZBP1/RIPK3-mediated necroptosis [1], the lack of a marker for in situ detection of pMLKL-S345, has rendered it very difficult to truly determine the contribution of necroptotic cell death following viral infection. It was previously published that necroptosis plays a role in chronic obstructive pulmonary disease caused by cigarette smoke [69] and that syncytial viral infections, which induces bronchiolitis, leads to activation of necroptosis [70]. This suggests already that under certain conditions lung cells, including human airway epithelial cells, can die necroptotically. Specifically for Coronaviruses, involvement of ZBP1 or RIPK3 in SARS-CoV-2 infection has been deduced from the attenuated cytokine and chemokine production and reduced lung damage found in infected mice deficient for either ZBP1 or RIPK3 [71]. Importantly, *Mlkl*^−*/−*^ animals do not present the same attenuation and appear to be more similar to control mice raising questions with regards to the exact role of necroptosis execution in SARS-CoV-2-infection-mediated systemic inflammatory syndrome [71]. While necroptosis was recently deemed dispensable from the pathogenesis of SARS-CoV-2 [72], it is not yet clear what cell death modality is truly responsible for the consequent acute respiratory disease syndrome which might engage into untoward necroptosis activation. Our data show that in lung sections obtained from SARS-CoV-infected mice, alveolar cells as well as other cells of different origin in the lung epithelium are positive for pMLKL-S345, suggesting necroptotic execution potentially in both infected and non-infected cells. This is in agreement with the data shown in Lagunas et al. [44], where c-Casp3 and TUNEL staining highlighted the involvement of cell death pathways other than apoptosis. It remains to be determined whether necroptosis activation is involved in the pathogenesis of development of severe disease following SARS-CoV infection or whether this is merely secondary cell death as a consequence of initial apoptosis without impact on disease severity. Considering the therapeutic opportunity to inhibit necroptosis pharmacologically, in the light of these results, the analysis of MLKL activation following viral infections utilising this novel detection protocol warrants further investigation.

The pro-apoptotic ability of RIPK3 was previously reported, in particular with regards to specific conformational changes that would promote Casp8 activation [55, 73]. This evidence has questioned whether the amelioration provided by *Ripk3*^*−/−*^ animals in different disease models should be immediately and solely connected to inhibition of necroptosis or/and also to its role in mediating inflammatory signals that drive apoptotic cell death. The fact that, in certain models, the outcome of MLKL deficiency is different from that of RIPK3 deficiency implies that the role of RIPK3 is context-dependent whereby RIPK3 can both support apoptosis and also drive necroptosis. The data obtained by the in situ detection of pMLKL-S345 in the skin and spleen of the *Shpn*^*m/m*^ and the *Shpn*^*m/m*^; *Ripk3*^−/−^ mice suggest that, in a tissue-dependent manner RIPK3 can either support apoptosis leading to secondary necroptosis (as observed in the skin) or drive necroptosis which then leads to secondary apoptosis (as observed in the spleen). Similarly, the data obtained in the SIRS model conclusively clarifies that the initial modality of cell death activated in response to TNF-induced shock is apoptosis. Our data suggest that any amelioration provided by RIPK3 or MLKL deletion is either due to prevention of RIPK3-mediated activation of Casp8 or to prevention of secondary necroptosis that might further contribute to a wave of systemic inflammation in these mice. In agreement with the study conducted by Newton et al. [55], we also find that TNF-induced mortality in C57BI/6N is predominantly driven by apoptosis-induced intestinal damage. Differently from C57BI/6J [57], we do not observe biologically significant levels of necroptotic cell death in the liver which was shown to be the main driver of TNF-induced mortality in this background strain. It was previously shown that C57BL/6J are exquisitely sensitive to TNF-induced shock than C57BL/6N due to presence of several passenger mutations in C57BI/6N [74], suggesting that these different backgrounds differentially influence the organ susceptibility of the mice to TNF-induced shock.

This work provides a protocol for the specific and sensitive detection of pMLKL-S345 in situ. Thereby, we provide an important tool that, for the first time, allows to effectively and reliably distinguish necroptosis from other modalities of cell death in situ in different tissues. Moreover, this work provides evidence of substantial differences between the in situ detection of different cell death markers, demonstrating the necessity to detect different modalities of cell death utilising molecular signatures in situ. Our protocol allows to not only distinguish these pathways from one another, but also to characterise which cell types, in different tissues, succumb via different modalities of cell death respectively in a given in vivo scenario. The ability to detect pMLKL-S345 allows to specifically determine the role of necroptosis in the pathology of many inflammatory disease models, importantly, without the need to delete FADD or Casp8 which is also known to create de novo roles of necroptosis not observed in their presence. Moreover, the in situ detection of pMLKL-S345 grants the ability to distinguish the pro-apoptotic role of RIPK3 from its role in activating MLKL as well as to decipher which initial cell death pathway promotes systemic inflammation by leading to secondary cell death activation. The ability to concomitantly detect pMLKL-S345, c-Casp3 and TUNEL shall contribute to determining which cell death pathway is responsible for initiating different inflammatory processes. This, in turn, shall provide the possibility to intervene therapeutically by specifically inhibiting the cell death modality or modalities responsible for a given pathology driven by inflammatory cell death.

### Supplementary information

Supplementary Material and Methods

Supplementary Figure 1

Supplementary Figure 2

Supplementary Figure 3

Supplementary Figure 4

Supplementary Figure 5

Supplementary figure Legends

## Data Availability

No datasets were generated or analysed during the current study. All raw histological images are available upon request.
