# Supplementary material for: The importance of murine phospho-MLKL-S345 in situ detection for necroptosis assessment in vivo
Source: Cell Death Differ. 2024 May 23;31(7):897–909. doi: 10.1038/s41418-024-01313-6 (PMC11239901; doi:10.1038/s41418-024-01313-6)
Supplement: Supplementary file 1 — Supplementary Material and Methods [file 41418_2024_1313_MOESM1_ESM.pdf]

Experimental Procedure

2.1 Sample preparation

Biopsy harvesting: Humanely sacrifice mice once reached endpoint criteria. Collect the mouse skin samples by harvesting skin biopsies from the dorsomedial region of the back of the mouse. Using a razor or a trimmer, remove as much hair as possible and with the scissors cut skin biopsies into a rectangle shape with the dimensions of 10 mm (L) x 5mm (W) so that the long side of the rectangle follows the Anterior-Posterior (AP) axis of the mouse (**2.1.1**). For colon and small intestine Swiss-rolls, longitudinally cut the respective tissues and wash with PBS to remove faeces. Roll up the intestinal samples from proximal to distal in a Swiss-roll shaped formation. For spleens, lungs and livers carefully extract and clean them up from any leftover adjacent tissue and wash them in PBS. Place the biopsies in an embedding cassette. Place the cassettes in 10% buffered formalin/ 4% Formaldehyde to fix the tissue overnight (O/N), *i.e.* for 8-12 hours (**2.1.2**). Store the cassettes containing the skin biopsies in 70% EtOH and the cassettes containing spleen, lung, liver and intestinal samples in PBS at 4°C at least for 24 hours before further processing (**2.1.3**).

Notes: (**2.1.1**) Make sure the rectangle-shaped biopsy is collected from the upper back of the mouse. Keep the area of harvesting consistent for all mice to obtain comparable results. (**2.1.2**) The skin specimens should be fixed O/N in formalin but it should not be kept for more than 24 hours in fixative. Over-fixation of samples may destroy the antigen site and interfere with the quality of the immunohistochemical staining. On the other hand, insufficient fixation may lead to the production of poor-quality paraffin blocks affecting the specimen cutting and its adherence to the slide during the retrieval step. (**2.1.3**) In general it is recommended to proceed with the embedding and cutting of the samples 24 hours following transfer into 70% EtOH or PBS. Please be mindful that extended storage times may harden the tissue which may negatively impact the preparation of the specimen for staining.

2.2 Tissue processing

Fixed tissues were paraffin embedded using the standard automatic tissue embedding protocols of the Vacuum tissue processor, Leica Biosystems, Model: ASP200S.

1. Transfer the cassettes containing the tissue sections to a Tissue Processor for dehydration. The skin tissues are processed in 70%, 80%, 96% EtOH for 2 hours each, 100% EtOH three times for 2 hours each and then in Xylol 2 times for 2 hours each. Spleen, lung, liver, colon and small intestine samples are processed in 30%, 50%, 70% EtOH for 2 hours each, 96% EtOH for 1 hour, 100% EtOH two times for 2 hours each and then in Xylol 2 times for 2 hours each; all steps are performed at RT. Skin tissues are then infiltrated in paraplast tissue embedding medium two times for 1 hour each and one last time for 2 hours, while the rest of the tissues two times for 1 hour each and a 2 hour incubation in between. All steps of paraffin infiltration are performed at 62°C.
2. In a Heated Paraffin Embedding Station, select the appropriate mold size. Pour the paraffin-embedding medium into the mold until one quarter of the mold is filled. Directly transfer the paraffin-infiltrated tissue inside the mold using heated tweezers. For skin sections make sure that the long side of the rectangle-shaped section is vertical to the mold's surface and in parallel to the mold's longer side. Hold the tissue in the centre and at the bottom of the mold using the tweezers to maintain its proper orientation

(2.2.1). Place the labelled lid of the sample cassette on top of the mold and slowly pour liquid paraffin into the mold until it is completely filled. Transfer the mold on a Cold Plate at -5°C for approximately 15 minutes until the paraffin is solid and hard enough to remove the block from the mold. The blocks are then placed on the Cold Plate until they are cut.

3. On a Microtome, trim the paraffin-embedded blocks by cutting thick sections of 20 μm until the complete surface of the embedded tissue is revealed. Set the cut size at 3 μm (lung, liver, colon, small intestine) or 5 μm (skin, spleen) and cut consecutive slices according to the number of sections needed.
4. Transfer the slices, grouped at a ribbon-shaped form of 6-8 slices, in a histological water bath for tissue sections set at 40°C for approximately 1-2 min. Pick every single floating section onto a correctly labelled Polysine Adhesion Slide. Transfer the slides on a Slide Dryer set at 40°C until the slides are dried and moisture-free. Continue the slide drying O/N in an incubator at 40°C **(2.2.2)**.

Notes: (**2.2.1**) Make sure all biopsies are placed in the mold in the correct orientation. This step will determine the final orientation of the biopsy in the paraffin block. This will ensure the produced cuts provide with the appropriate view of: the interfollicular epidermis, the hair follicles and hair orientation when stained for skin, white and red pulp for spleen, bronchioles, alveoli and veins for lungs, the crypts, villi and lumen for intestinal samples (**2.2.2**). The drying step is critical to ensure proper adhesion of the tissue on the slide. Insufficient drying of the sections may lead to tissue loss during the staining procedure and especially during Heat-induced Retrieval.

2.3 Staining

1. **Deparaffinisation/Rehydration:** Incubate the slides in Xylol or Xylene substitute three times for 5 minutes each, twice in 100% v/v ethanol for 5 minutes each, 95% v/v ethanol in water for 5 minutes, 70% v/v ethanol in water for 3 minutes, 50% v/v ethanol in water for 3 minutes and lastly two times in deionised (DI) water (diH_2O) for 5 minutes each (**2.3.1, 2.3.2**).

2. **Antigen Retrieval:** Prepare fresh 1x Dako Target Retrieval Solution Buffer and add 0.05% Tween20. Transfer the slides in a Slide holder and then in a Staining Dish or directly in a Coplin jar containing the retrieval buffer. Incubate the slides in the retrieval buffer for 10 minutes at room temperature. Fill the Pressure Cooker with 500-1000 ml of ddH_2O and place the Dish/Coplin jar inside the cooker. Choose the High-Pressure program (114°-121°C) and set the timer at 13 minutes (**2.3.3, 2.3.4, 2.3.5**). Directly after the end of the program release the pressure, remove the lid from the Pressure cooker and let the slides sit in the same buffer for around 1 hour to allow the buffer progressively cool down. Wash the slides in diH2O twice for 5 minutes each and air-dry them carefully. Using a Hydrophobic barrier pap pen, draw a circle around each tissue section (**2.3.6**).

3. **Blocking and Primary Antibody Incubation:**

   i. **Endogenous Peroxidase Blocking:** Block endogenous peroxidase activity by incubating the slides in BLOXALL Blocking solution for 10 minutes at RT in the dark by adding the reagent to each tissue section dropwise. Immediately rinse the slides in diH_2O and incubate them for 5 minutes in washing buffer (henceforth referred as WB, see Supplementary Table 2).

ii. **Protein Blocking:** Block the antibody non-specific binding sites by incubating the tissue sections in animal free serum RTU for 30 minutes at RT. Wash the slides in WB for 5 minutes.

iii. **Biotin/Avidin Blocking:** Block avidin binding sites by incubating the tissue sections in Avidin blocker for 5 minutes. Wash the slides in WB for 5 minutes and block endogenous biotin and biotin receptors by incubating the tissue sections in Biotin Blocker for 5 minutes. Wash slides in WB three times for 5 minutes each followed by a quick rinse in TBS.

iv. **Lambda Protein Phosphatase (Lambda PP) Treatment**
Prepare Lambda PP working solution by diluting the supplied 10x concentrated NEBuffer Pack for Protein MetalloPhosphatases (PMP) and $MnCl_2$ in diH_2O. Vortex and add Lambda PP to a final concentration of 40.000U/ml (**2.3.7**). Include internal controls by treating slides with the prepared 1x Lambda PP Buffer only. Incubate slides at 37°C in a wet chamber for 24h (**2.3.7**). Wash slides thoroughly for three times in WB for 5 minutes each followed by a quick rinse in TBS.

v. **Primary Antibody Incubation:** Apply the primary pMLKL-S345 antibody at a final dilution of 1:2000 in 0.1% v/v Triton-X, animal free blocker and incubate them overnight at 4°C in a wet chamber filled with water (**2.3.8**).

4. **Secondary Antibody Incubation:** Wash the slides in WB, three times for 10 minutes each on a shaker at 200 rpm and then quickly rinse in TBS. Incubate the tissue sections in Biotinylated Goat Anti-Rabbit IgG Antibody (H+L), by diluting it 1:200 in 0.1% v/v Triton-X, animal free blocker for 40 min.

   For the immunohistochemical assay: In the mean-time, prepare the ABC Reagent: Add 1 drop of Reagent A (Avidin, ABC Elite) in 5 ml of 1xTBS in a 15 ml falcon tube and vortex thoroughly. Add 1 drop of Reagent B (Biotinylated HRP, ABC Elite) in the same tube and mix well. Let the reagent stand at room temperature for at least 30 minutes before use. Wash the slides three times for 5 minutes in WB on the shaker and quickly rinse them in TBS. Add the ABC reagent in each tissue section dropwise and incubate them for 40 minutes. Wash the slides three times for 5 minutes in WB on the shaker and then wash the slides in diH_2O for 5 minutes.

5. **DAB staining, counterstaining and mounting:** Combine 1 drop (~30 µl) of ImmPACT DAB Reagent to 1 ml ImmPACT DAB Diluent and apply the DAB mixture to each tissue section and incubate. Stop the reaction by dipping the slide in water when the signal staining reaches the desirable intensity. Then, rinse twice with diH_2O (**2.3.9, 2.3.10**).

   For Hematoxylin counterstaining, prepare two chambers, one containing hematoxylin and the other one containing tepid tap H_2O. Transfer the slides in a slide basket/rack and dip it in hematoxylin for 2 min. Transfer the slide basket in the chamber containing tepid tap H_2O and wash the slides by moving the slide basket up and down for approximately 10 seconds. Rinse well in a container under running tap H_2O for approximately 20 sec and let the slides stand in tap water for 15 minutes, until Haematoxylin acquires a bluish tint.

   Dehydrate the tissue sections by dipping the slides in 70%, 95%, 100% ethanol and Xylene for 1 minute each.

Mount the slides by adding 1-2 drops of Mounting Medium and cover the slides with a coverslip (**2.3.11**) before letting them dry O/N at RT.

For the Immunofluorescence assay: After incubating the slides in Biotinylated Goat Anti-Rabbit IgG Antibody (H+L), wash the slides three times for 5 minutes in WB on the shaker and quickly rinse them in TBS. Incubate the tissue sections in Alexa Fluor 594 Streptavidin (1:200) and DAPI at a final concentration of 0.1-1 µg/ml in animal free blocker for 30 minutes.

Wash the slides thoroughly three times for 10 minutes each in WB in the dark on the shaker and then rinse the slides in diH_2O for 5 minutes. Mount slides in anti-fade mounting media and store at 4°C for at least 24 hours before visualization under the confocal microscope.

Notes: (**2.3.1**) Always work under a fumes' hood when using xylene to avoid its hazardous effects. (**2.3.2**) In case of incomplete deparaffinisation, incubate the slides at 60°C for 20 minutes prior to xylene incubation. (**2.3.3**) The time, temperature and pH of the retrieval buffer should be kept consistent to properly retrieve the antigenic site and avoid either inadequate retrieval or destruction of the antigenic site. This will prevent loss of signal or non-specific antibody binding and any possible background staining. (**2.3.4**) Alternatively, heat the slides in the Coplin jar in the microwave oven at half power (350 W) until the buffer starts to boil. Continue boiling at 120 W for 15 minutes. At this point, make sure to replenish retrieval buffer when you observe spillage from the Coplin jar to avoid drying the tissue. Remove the jar from the microwave oven and let the slides sit at room temperature (RT) in the same buffer for 40 minutes until the temperature reaches around 50°C. **(2.3.5)** It is often experienced that during retrieval time, tissues of different origin and age might detach from the slides. To prevent this from occurring while ensuring correct retrieval, we recommend the assembly of a "sandwich" cassette. Briefly, add retrieval buffer to a slide of interest. Place a coverslip on top of the slide and then add retrieval buffer to the coverslip. Gently place the second slide face down on top of the coverslip and secure the sandwich-like cassette with metal clips. This will apply a mechanical compression to the two tissue sections to prevent detachments and preserve tissue morphology while allowing adequate epitope retrieval. Add the sandwich into a Coplin jar and proceed with boiling into a pressure cooker for 10-13 minutes. Follow procedure as described above. (**2.3.6**) Carefully circle the tissue sections around the edges using the pap pen to prevent diffusion of the incubation buffers and reagents. Avoid drawing the circle too close to the tissue sections as this might prevent liquid diffusion over the section due to hydrophobic tension. (**2.3.7**) Concentration of Lambda PP, temperature and incubation times were optimised for skin tissue sections for this staining assay. Since this de-phosphorylation assay varies with tissue samples and temperature, we recommend you optimise these parameters for different incubation times or tissue samples. (**2.3.8**) Primary antibody dilution has been optimised for skin, spleen, lung, liver and intestinal sections. We recommend to titrate the dilution for other organs in the range of 1:1000-10000 to find optimal antibody concentration to better assess specific pathological conditions of interest or in case of high Noise/ Signal ratio. (**2.3.9**) During DAB exposure, observe the slides under the microscope until the desirable intensity is reached. Stop the reaction simply by dipping the slides in water. The reaction usually takes 15 seconds for optimal intensity (**2.3.10**) Always use a positive and a negative control to validate the performance of your staining. (**2.3.11**) To prevent any bubbles from being trapped between the coverslip and the slide, add the mounting medium on a wet slide, directly after the incubation step in xylene. Place the coverslip in a 60° angle until it touches one side of the slide and slowly release it on top of it by excreting mild pressure with an angled tweezers. To remove any bubbles that were still trapped, mildly exert pressure

on top of the slide using the tweezers. Be careful as too much pressure might destroy the tissue.

2.4 Imaging Analysis

Immunohistochemistry slides were digitalised using a digital slide scanner (NanoZoomer S360MD Slide scanner system, Hamamatsu Photonics K.K.) with a 40x magnification lens. The acquired brightfield images were analysed in QuPath, an open-source software [1]. Additional 10x and 63x pictures were acquired with Leica DM750 Binocular microscope. The immunofluorescence pictures were acquired with LSM 980 with Airyscan 2, 20x and 63x lens, Zeiss at the CECAD Imaging Facility, University of Cologne.

1. Create Pixel Classifier

In QuPath, to quantify the positively stained cells, we generated, first and when necessary, a pixel classifier in order to exclude the areas of the tissue that should not be accounted for during the quantification step and would otherwise interfere with the "positive cell detector". These areas mainly comprised of the hair follicles since the melanin from the hair can be considered falsely as positive signal by the automated detector leading to false positive results.

1. On QuPath main window, create a new project by clicking on 'Create Project'. Select the directory you want to save your project to.
2. Click on 'Add images' and import the .dnpi files by drag and drop or simply by clicking on 'Add Image'. Set image type "Brightfield (H-DAB)" and click "Import".
3. To generate a pixel classifier, you need first to create a training image.
4. Click Classify>Training Images>Create region annotations. Set width and height at 150, Size units: µm, Classification: Region* and click on 'Create region' 5-10 times. Place the square training regions on representative areas that include both areas that should be classified and areas that should be excluded from the classification. Before you continue make sure to save your project (File>Save).
5. Click Classify>Training images>Create training image. Set Classification: Region*, leave the rest as default and click "OK". A new composite image will be generated at the end of your project by combining the different numbers of region annotations you generated. If required, set image type as 'Brightfield (H-DAB)' and click "Apply".
6. Select the brush tool and draw on the areas with positive cells that should be classified. On the left side of the main window click on "Annotations". You will find listed all the annotations you generated with the brush tool. In the Annotations panel select the annotations you generated, in the class panel right next to the list with the annotations, click on 'Positive'>Set Class. Repeat the same procedure, this time creating annotations that contain only areas that should be excluded from the classification (i.e., the hair). Set Class "Negative". (In the "Class" Panel you can add or remove Classes by clicking at the three dots "More options").
7. Click on Classify>Pixel classification>Train pixel classifier (Ctrl + shift + P).
8. Select Classifier: "Random Trees (RTrees)", Resolution: "Very high (0.46 µm/px)", leave rest as default and click on "Live prediction".
9. The Pixel Classifier might need further optimisation. Adjust the Pixel Classifier options until best results are reached, add "Classifier name" and click on "Save".

2. Create Regions Of Interest (ROIs) and Run Pixel Classifier

1. In the "Project" tab select the file you want to work with.

2. Click on Analyze>Deprecated>Simple Tissue Detection (deprecated). This will generate automatically annotations for the tissue sections based on a chosen threshold.
3. In the “Annotations” tab you will find the newly generated Annotations.
4. Select all the annotations in the “Annotations” tab and go to Classify>Pixel Classification>Load pixel classifier. Choose model: Select the classifier you generated, Region: “Any annotation ROI” and click on “Create objects”.
5. A new “Pixel Classifier” window will pop up. Choose parent objects: “Current selection” and click “OK”.
6. In the “Create objects” window, leave everything as default and click “OK”.
7. For every annotation two new annotations will be generated, one classified as positive and one as negative.

3. Positive cell detection and counting

1. Click on “Annotation (Positive)” in the annotation tab. Go to Analyze>Cell detection>Positive cell detection.
2. In the “Positive cell detection” window that pops up, set the parameters as desired and press “Run”. Detection annotations will be generated. By looking at them you can decide to change accordingly the “Positive cell detection” parameters and re-run the detection. In principle, we suggest you optimize your detection method by mainly trying and adjusting the “Intensity Parameters”, and “Intensity Threshold Parameters”.
3. Go to Measure>Show annotation measurements. Right click on the “Annotation results” window>Show classes>Positive. Here you will find the quantification results (including the total number of cells, the number of cells stained positive and the percentage of the positive cells).

2.5 Graph and Statistics

Plot the percentages of positive cells per total number of cells for each section or image for the different biological replicates were in a statistical software. Perform two-way Anova statistical analysis to determine significance among the different groups (P values=0.05 *; 0.01**; 0.001*** 0.0001****).

Supplementary Table 1

Reagents, materials and the equipment necessary for the execution of the protocol are given in Table 1. The recipes for the preparation of the reagents can be found in the Reagents section (Table 2).

Resources	Source	Catalogue number
**Antibodies**		
Phospho-MLKL (Ser345) (D6E3G)*	Cell Signalling Technology	#37333
**Kits/ Assays**		
Goat Anti-Rabbit IgG Antibody (H+L), Biotinylated, 1.5 mg*	Vector Laboratories	#BA-1000
VECTASTAIN Elite ABC-HRP Kit, Peroxidase (Standard)*	Vector Laboratories	# PK-6100
ImmPACT DAB Substrate Kit, Peroxidase (HRP)	Vector Laboratories	#SK-4105

Alexa Fluor 594 Streptavidin	Thermo Fisher Scientific	# S11227
Animal-Free Blocker and Diluent, R.T.U.	Vector Laboratories	#SP-5035-100
Avidin/Biotin Blocking Kit*	Vector Laboratories	# SP-2001
BLOXALL Endogenous Blocking Solution, Peroxidase and Alkaline Phosphatase	Vector Laboratories	#SP-6000-100
Hematoxylin Counterstain	Vector Laboratories	#H-3401
Hematoxylin Counterstain	Carl Roth	# T865.3
Eosin	Carl Roth	# X883.2
VectaMount Express Mounting Medium	Vector Laboratories	#H-5700
ProLong Gold Antifade Mountant	Thermo Fisher Scientific	# P36930
DAPI, dilactate	Sigma-Aldrich	# D9564
Dako Target Retrieval Solution, pH 9 (x 10), Concentrate*	Agilent Technologies	#S236784-2
Lambda Protein Phosphatase*	New England Biolabs (NEB)	#P0753S
**Chemicals**		
Xylol (Isomere)<br>ROTI Histol (Xylene substitutes)	Carl Roth<br>Carl Roth	#9713<br>#6640
Xylene (mixture of isomers) ≥98.5%, AnalaR NORMAPUR ACS, Reag. Ph. Eur. analytical reagent (for Tissue Processor)	VWR Chemicals	#28975.360
Ethanol ROTIPURAN ≥99.8%, p.a.	Carl Roth	#9065
ETHANOL 96 % DENATURED WITH MEK, IPA AND BITREX® (MIN. 96.0 %)	TH.GEYER	#11832327
Ethanol denatured ≥99.5%, GPR RECTAPUR® (denatured with 1% MEK) (for Tissue Processor)	VWR Chemicals	#85033.360
Surgipath Paraplast	Leica Biosystems	#39602012
Tween-20	Sigma-Aldrich	#P7949-2.5L
Triton X-100	Sigma-Aldrich	#X100
Sodium chloride 99.5-100.5%, AnalaR NORMAPUR® ACS, Reag. Ph. Eur. analytical reagent, NaCl	VWR Chemicals	#27810.364
Trizma base, ary Standard and Buffer, ≥99.9% (titration), crystalline, $NH_2C(CH_2OH)_3$	Sigma-Aldrich	#T1503
EDTA disodium salt dihydrate 99.0-101.0%	VWR Chemicals	# 20302.293

Formalin solution, neutral buffered, 10%	Sigma-Aldrich	#HT501128-4L
Formaldehyde solution, phosphate buffered, 4%	Walter CMP GmbH	# WAL60622
**Treatments**		
LPS-free mTNFα	BioLegend	#575208
Tamoxifen	Sigma-Aldrich	#T5648
**Equipment**		
Vacuum tissue processor	Leica Biosystems	Model: ASP200S
Modular Tissue Embedding Center	Leica Biosystems	Model: EG1150
Fully Automated Rotary Microtome	Leica Biosystems	Model: RM2255
Cold Plate for Modular Tissue Embedding System	Leica Biosystems	Model: EG1150C
Water Bath for Paraffin Sections	Leica Biosystems	Model: HI1210
Flattening table for clinical histopathology	Leica Biosystems	Model: HI1220
TintoRetriever Pressure Cooker	BioSB	#BSB7008
Microwaves	Bomann	#MW6014CB
Shaker	Grant Instruments	Model: PMR-30
Confocal Microscope	ZEISS	Model: LSM 980 with Airyscan 2
Light Microscope	Leica Biosystems	Model: DM750
Slidescanner for brightfield images	Hamamatsu	#C13220-21MDEU
**General Supplies**		
Staining trough acc. to Coplin*	Carl Roth	#YC09.1
Polysine Adhesion Slides	Epredia	#J2800AMNZ
Tissue embedding cassettes	Kabe Labortechnik	#054681
Cover Glasses	DWK Life sciences	#235505508
Hydrophobic Barrier PAP Pen	Vector Laboratories	#H-4000
Stain Tray	Simport Scientific	#M918-2
Set for histology and cytology with 12 dishes	Bio Optica	#10-10
Plastic Staining Dish	BioSB	#BSB 7009
Slide Holder (24 Slides)	BioSB	#BSB 7010
Staining dish with attached lid	Bio Optica	#10-30
Plastic slide basket	Bio Optica	#10-42
Disposable Blades	Leica Biosystems	#14035838382
**Software**		
QuPath (Open Software for Bioimage Analysis)		https://qupath.github.io/
Prism - GraphPad		https://www.graphpad.com/features

* We suggest that the indicated reagents and materials are not replaced to ensure the highest possible experimental performance. The rest of the reagents, materials and general supplies could be replaced based on the availability and capacity of the respective consumables.

Supplementary Table 2

Reagent Preparation

"Homemade" Tris-EDTA Antigen Retrieval Buffer at pH 9, 1x

**Tris-EDTA Retrieval Buffer**	**Amount**	**Final Concentration**
Trizma base	1,21g	10mM
EDTA	0,37g	1mM
HCl	Until pH 9	n/a
H_2O	1L	n/a
Tween-20	0,5ml	0,05% (v/v)

To prepare 1L of 1x Tris-EDTA Antigen Retrieval Buffer stock solution, dissolve the indicated amount of Trizma and EDTA in 900ml of double-distilled water (ddH_2O). Adjust the pH at 9 if necessary and fill up to 1L with ddH_2O. Store the Buffer at 4°C for long-term use.

TBS 10x Concentrated

**TBS**	**Amount**	**Final Concentration**
Trizma base	24,2g	200mM
NaCl	87,75g	1.500mM
HCl	Until pH 7,4	n/a
H_2O	1L	n/a

To prepare 1L of 10X TBS stock solution, dissolve the indicated amount of Trizma and NaCL in 900ml of double-distilled water (ddH_2O). Adjust the pH at 7.4 with 12N HCl and fill up to 1L with ddH_2O. TBS is stable at 4°C for 3 months. To make 1x TBS working solution, dilute 100ml of 10x TBS in 900ml of ddH_2O. Store the TBS solutions at 4°C.

Washing Buffer (WB), 1x

**TBS-T 0.1% (v/v)**	**Amount**	**Final Concentration**
1x TBS	1L	n/a
Tween-20	1ml	0,1% (v/v)
Total	n/a	1L

The washing buffers should be prepared freshly on the day of use. Store them at 4°C for short-term use.
