# Supplementary figures and images for: The importance of murine phospho-MLKL-S345 in situ detection for necroptosis assessment in vivo
Source: Cell Death Differ. 2024 May 23;31(7):897–909. doi: 10.1038/s41418-024-01313-6 (PMC11239901; doi:10.1038/s41418-024-01313-6)

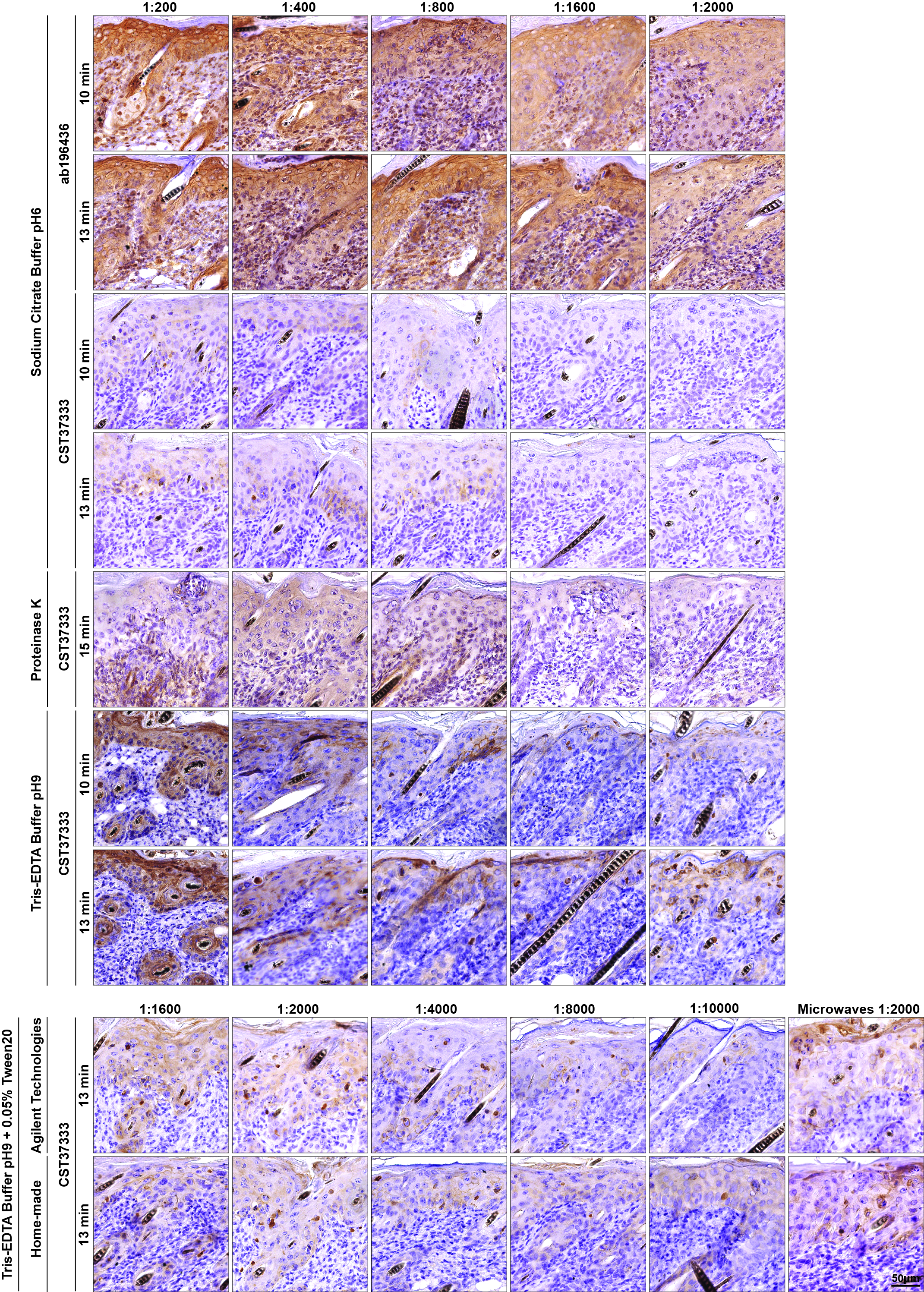

Supplement: Supplementary file 2 — Supplementary Figure 1 [file 41418_2024_1313_MOESM2_ESM.jpg]

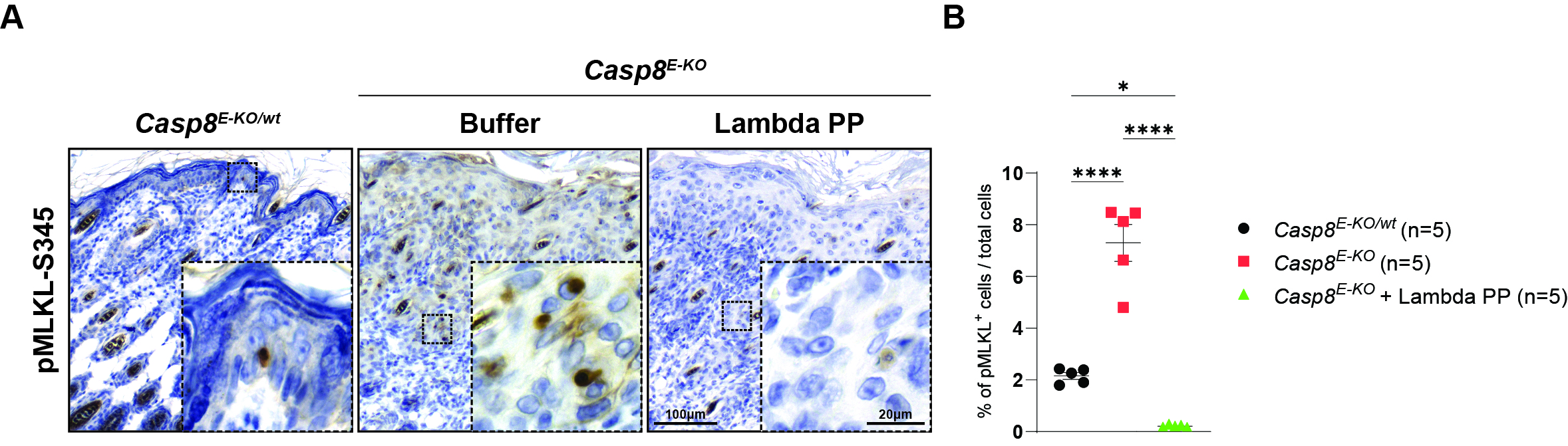

Supplement: Supplementary file 3 — Supplementary Figure 2 [file 41418_2024_1313_MOESM3_ESM.jpg]

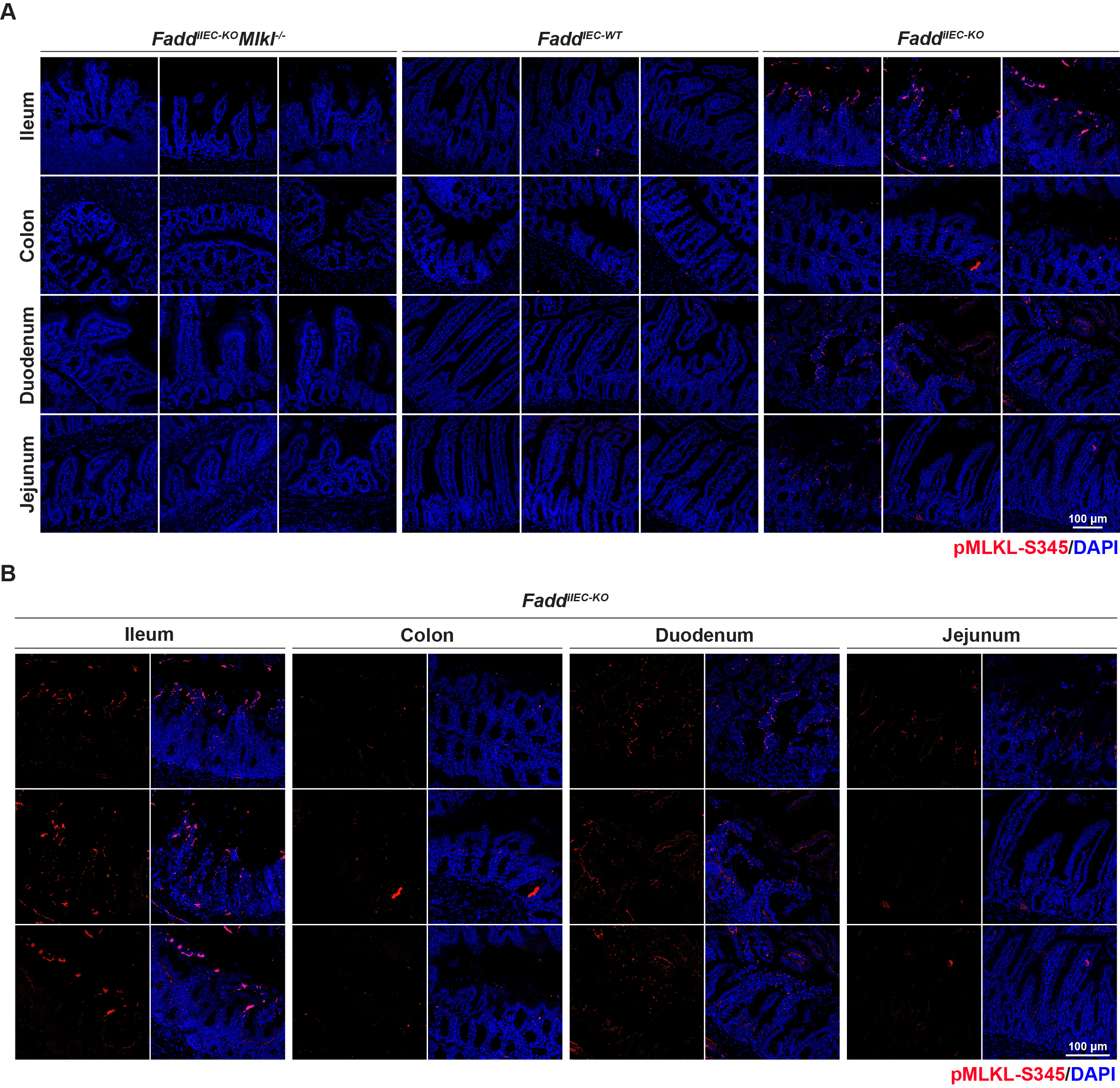

Supplement: Supplementary file 4 — Supplementary Figure 3 [file 41418_2024_1313_MOESM4_ESM.jpg]

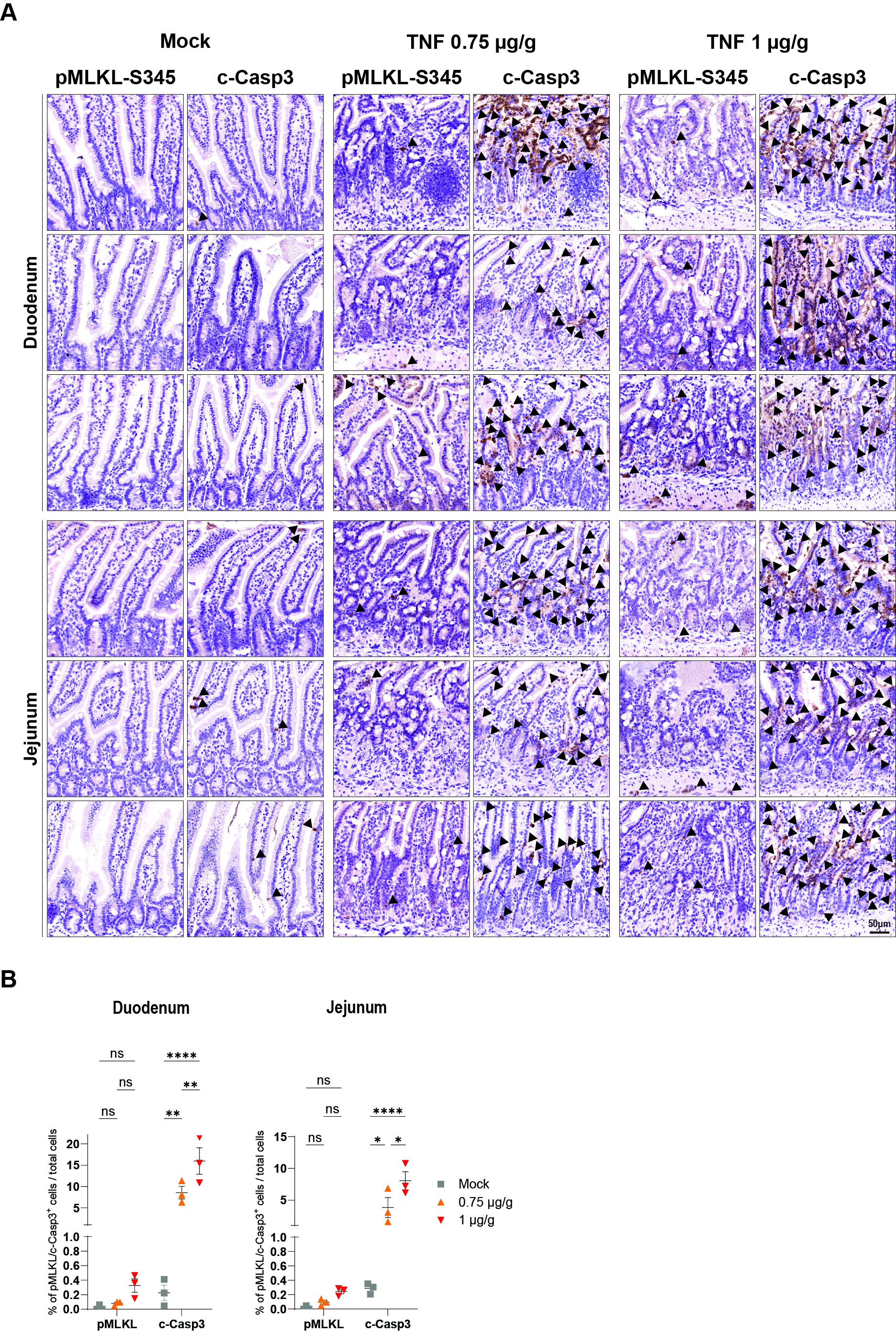

Supplement: Supplementary file 5 — Supplementary Figure 4 [file 41418_2024_1313_MOESM5_ESM.jpg]

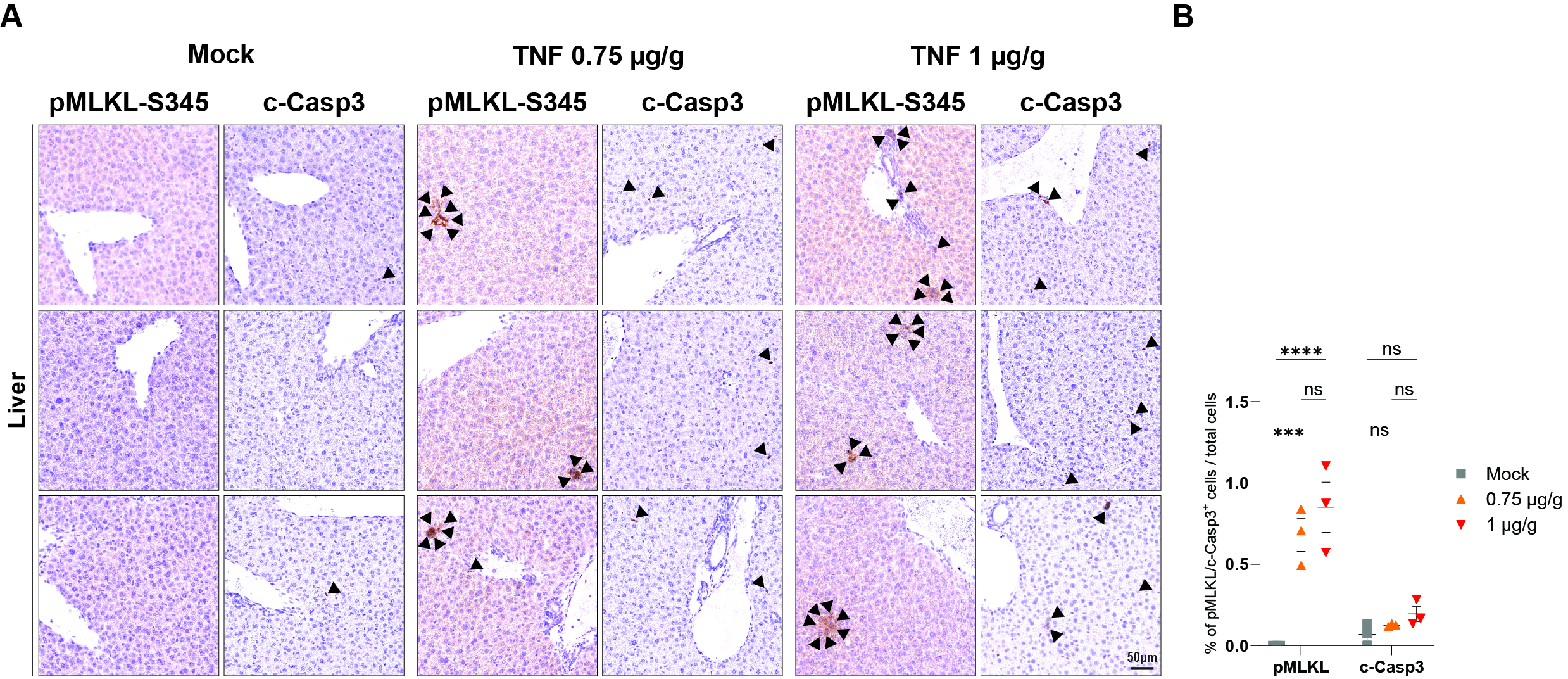

Supplement: Supplementary file 6 — Supplementary Figure 5 [file 41418_2024_1313_MOESM6_ESM.jpg]
