# Supplementary material for: The importance of murine phospho-MLKL-S345 in situ detection for necroptosis assessment in vivo
Source: Cell Death Differ. 2024 May 23;31(7):897–909. doi: 10.1038/s41418-024-01313-6 (PMC11239901; doi:10.1038/s41418-024-01313-6)
Supplement: Supplementary file 7 — Supplementary figure Legends [file 41418_2024_1313_MOESM7_ESM.docx]

**Supplementary Figure 1:** Representative images of skin sections from *Casp8^E-KO^* mice immunostained with pMLKL-S345 with the indicated α-pMLKL-S345 antibodies, antibody dilutions, retrieval method/retrieval buffer and retrieval times. Slides were digitalised in a digital slide scanner and images were acquired in QuPath. Scale bar: 50µm.

**Supplementary Figure 2:** **(A)** Representative images of skin sections from *Casp8^E-KO^* mice treated with 40.000U/ml Lambda Protein Phosphatase (Lambda PP) in Lambda PP buffer and immunostained with pMLKL-S345. Control slides of skin section from *Casp8^E-KO^* mice treated with Lambda PP buffer and *Casp8^E-KO/wt^* mice were also immunohistochemically assessed (n=5 in each group). Scale bars: 100µm (representative field) and 20µm (magnified selcted areas). **(B)** Graph showing quantification of pMLKL-S345 immunostaining obtained via QuPath after slides were digitalized in a digital slide scanner as described in the supplementary methods section. Total skin sections were analysed and total numbers of cells were obtained to calculate the percentage of positive cells over the total amount of cells detected. Data are presented as mean + SEM and each dot represents one mouse. P values were calculated via unpaired two-tailed t-test. **P≤0.01

**Supplementary Figure 3:** Co-housed 11-weeks old *Fadd^fl/fl^; Villin-Cre-ERT^2^* and *Fadd^fl/fl^; Mlkl^-/-^; Villin-Cre-ERT^2^* mice were injected with tamoxifen (10mg/mL) for 3 consecutive days to induce deletion of *Fadd* from intestinal epithelial cells (*Fadd^iIEC-KO^ and Fadd^iIEC-KO^; Mlkl^-/-^* respectively). Age-matched *Fadd^fl/fl^* mice treated with tamoxifen in the same way served as controls (*Fadd^iIEC-WT^*). **(A**-**B)** Representative images of ileum, colon, duodenum and jejunum sections (Swiss-rolls) from mice of the indicated genotypes at the endpoint (14 weeks) immunostained with pMLKL-S345 via immunofluorescence. Red dots indicate pMLKL-S345 signal. DAPI for nuclei. Scale bar: 100µm.

**Supplementary Figure 4:** Co-housed 10–12-week-old female C57Bl6/N wt mice were injected with 0.75µg/g or 1µg/g of ice-cold recombinant, LPS-free mouse TNF intravenously. Control mice received a corresponding volume of ice-cold LPS-free PBS. Mice were sacrificed 6 hours post-injection (n=3 in each group). **(A)** Representative images of duodenum and jejunum sections (Swiss-rolls) of wt mice immunostained with pMLKL-S345 and c-Casp3 as indicated. Scale bars: 50 µm. Arrowheads indicate pMLKL-S345-positive cells and c-Casp3-positive areas. **(B)** Graph showing quantification of illustrated pictures for each immunostaining. Total numbers were obtained to calculate the percentage of positive cells over the total amount of cells detected. Data are presented as mean + SEM and each dot represents one mouse. P values were calculated via two-way Anova, Tukey's multiple comparisons test. **P≤0.01, ***P≤0.001, ****P≤0.0001, ns: not significant.

**Supplementary Figure 5:** Co-housed 10–12-week-old female C57Bl6/N mice were injected with 0.75µg/g or 1µg/g of ice-cold recombinant, LPS-free mouse TNF intravenously. Control mice received a corresponding volume of ice-cold LPS-free PBS. Mice were sacrificed 6 hours post-injection (n=3 in each group). **(A)** Representative images of liver sections immunostained with pMLKL-S345 and c-Casp3 as indicated. Scale bars: 50 µm. Arrowheads indicate pMLKL-S345-positive cells or c-Casp3-positive cells. **(B)** Graph showing quantification of illustrated pictures for each immunostaining obtained via QuPath. Total numbers of cells were obtained to calculate the percentage of positive cells over the total amount of cells detected. Data are presented as mean + SEM and each dot represents one mouse. P values were calculated via two-way Anova, Tukey's multiple comparisons test. **P≤0.01, ***P≤0.001, ****P≤0.0001, ns: not significant.
